# Recent Progress in Designing Halide-Perovskite-Based System for the Photocatalytic Applications

**DOI:** 10.3389/fchem.2020.613174

**Published:** 2021-01-13

**Authors:** Yizhou Zhao, Lanning Wang, Tinglu Song, Alexander Mudryi, Yujing Li, Qi Chen

**Affiliations:** ^1^Beijing Key Laboratory of Construction Tailorable Advanced Functional Materials and Green Applications, Experimental Center of Advanced Materials, School of Materials Science and Engineering, Beijing Institute of Technology, Beijing, China; ^2^Scientific-Practical Material Research Centre of the National Academy of Science of Belarus, Minsk, Belarus

**Keywords:** halide perovskite, photoelectrocatalysis, HER, water splitting, CO_2_ reduction, photocatalytic organic synthesis, organic chemical degradation

## Abstract

The halide perovskite material has attracted vast attention as a versatile semiconductor in the past decade. With the unique advantages in physical and chemical properties, they have also shown great potential in photocatalytic applications. This review aims at the specific design principles triggered by the unique properties when employing halide-perovskite-based photocatalytic systems from the following perspectives: (I) Design of photoelectrocatalytic device structures including the n-i-p/p-i-n structure, photoelectrode device encapsulation, and electrolyte engineering. (II) The design of heterogeneous photocatalytic systems toward the hydrogen evolution reaction (HER) and CO_2_ reduction reaction, including the light management, surface/interface engineering, stability improvement, product selectivity engineering, and reaction system engineering. (III) The photocatalysts for the environmental application and organic synthesis. Based on the analyses, the review also suggests the prospective research for the future development of halide-perovskite-based photocatalytic systems.

## Introduction

**Figure 1:**
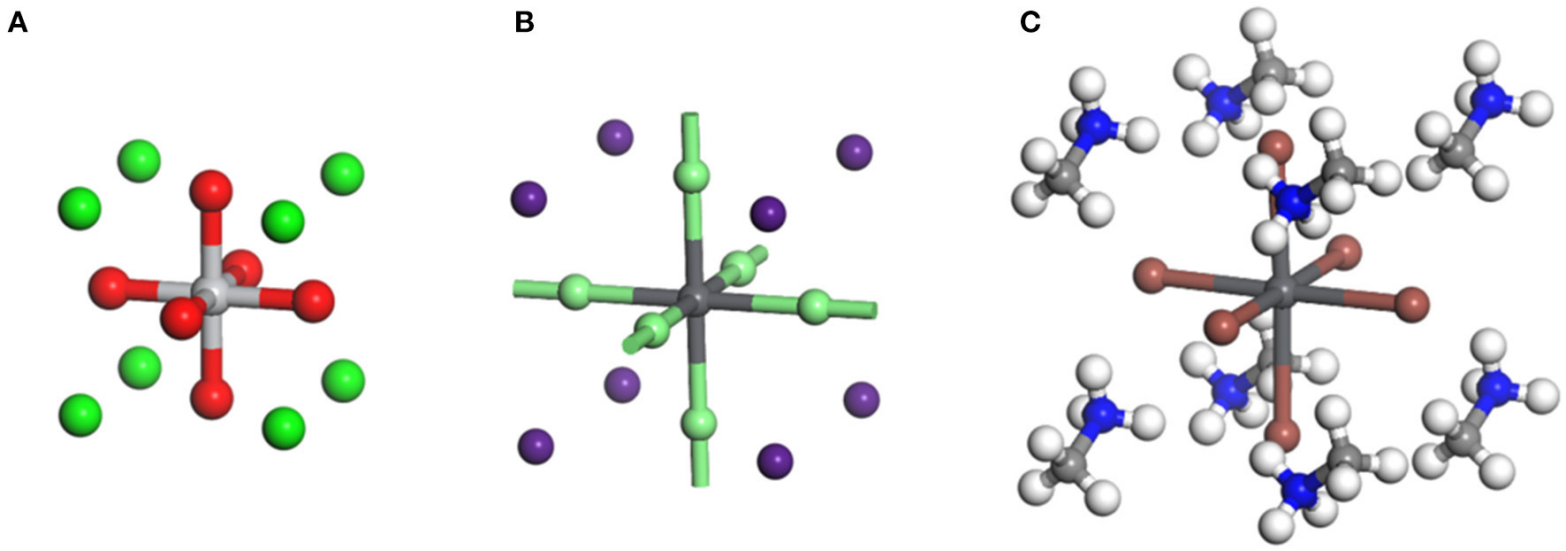
**(A)** Crystal structure of CaTiO_3_ perovskite. Green: Ti^4+^, red: O^2−^, gray: Ca^2+^ (Mahmood and Al-Shakarchi, 2011). **(B)** Crystal structure of inorganic CsPbBr_3_ perovskite. Light green: Br^−^, purple: Cs^+^, gray: Pb^2+^ (Sakata et al., 1979). (**C)** Crystal structure of organic–inorganic hybrid MAPbI_3_ perovskite. White: H, blue: N, light gray: C, dark gray: Pb^2+^, orange: I^−^ (Feng and Xiao, 2014).

The perovskite, previously referring to a particular mineral with the formula as CaTiO_3_ in a narrow sense, represents a family of material sharing the similar crystal structure. The ideal perovskite structure has an arrangement of ions that belong to the cubic structure, which is typified by SrTiO_3_ with a = 3.905 Å and *Z* = 1 belonging to the Pm-3m space group (Bhalla et al., 2000). The Ti^4+^ and Sr^2+^ ions occupy the corner and center positions of the cube, respectively. The oxygen ions are placed at the centers of the six faces, constructing corner-shared strings of TiO_6_ octahedra, which extend in three dimensions (Figure 1A).

The halide perovskite family represents a series of emerging semiconductor materials in recent years with the halide ions occupying the face-center positions as the name suggests (Stoumpos and Kanatzidis, 2016). Despite the similar structure, the halide perovskite differs from oxide perovskite by forming (PbX_6_) octahedra, which endows it with unique semiconducting properties (Yin et al., 2014b). The three-dimensional (3D) halide perovskite possesses an ABX_3_ atomic composition, wherein the A, B, and X can be selected from various types of ions, e.g., A = Cs^+^, Rb^+^, methylamine (MA^+^), formamidine (FA^+^); B = Pb^2+^, Sn^2+^; X = Cl^−^, Br^−^, I^−^ (Brivio et al., 2014). If the A site is occupied by inorganic ions such as Cs^+^ or Rb^+^, it is normally referred to as all-inorganic perovskite, whereas the material with the A-site being occupied by MA^+^ and FA^+^ ions is classified as

organic–inorganic hybrid perovskite (Figures 1B,C). The lead halide perovskites can also be prepared into two-dimensional (2D) structure by replacing the A-site group with a long-chain amine cation such as butylamine (BA), octylamine (OA), etc. (Lee et al., 2018). The intercalating molecules in 2D perovskite form natural quantum wells dividing the (PbX_6_) octahedron lattice into thin inorganic layers consisting of one (*n* = 1), two (*n* = 2), or three (*n* = 3) lead iodide layers (Ponseca et al., 2014). The periodic quantum wells generate a unique electronic band structure such as broadened band gap and hence improved moisture stability, which is different from the 3D structure (Wu et al., 2015).

The unique optoelectronic properties of the halide perovskites have been systematically investigated and reported, including their high defect tolerance (Kang and Wang, 2017), tunable band structure (Sutter-Fella et al., 2016), excellent photoluminescence (Zhu et al., 2015), spin-coating manufacture methods (Qaid et al., 2016), etc. Among their potential applications like solar cell (Kim et al., 2014; Mei et al., 2014; Yin et al., 2014a), light-emitting diode (LED) (Vassilakopoulou et al., 2017), field-effect transistor (Chin et al., 2015), and radiation detection (Stoumpos et al., 2013), the perovskite is receiving growing attention in their photocatalytic application for the green solar to chemical fuel conversion (Chen et al., 2019).

Photocatalytic and photoelectrocatalytic technologies are considered to be promising strategies to generate solar fuels and create new pathways of chemical synthesis (Reece et al., 2011; Garcia-Segura and Brillas, 2017; Kou et al., 2017; Sohn et al., 2017). Various types of materials have been reported as potential photocatalysts, such as metal–organic compounds (Narayanam and Stephenson, 2011), TiO_2_ (Zaleska, 2008), quantum dots (QDs) (Cheng et al., 2018), graphitic carbon nitride (g-C_3_N_4_) (Wen et al., 2017), etc. (Spasiano et al., 2015). More recently, numerous works using halide perovskite for photocatalysis have been reported (Chen et al., 2019; Liang et al., 2019; Huynh et al., 2020; Kim et al., 2020; Wang H. et al., 2020). Despite the excellent optoelectronic properties, the halide perovskite suffers some disadvantages such as the crystal structure instability (Chen et al., 2018), poor moisture tolerance (Smith et al., 2014), lack of catalytic active sites (Tang et al., 2019), and inefficient carrier separation for nanoscale unit (Jiang et al., 2015). Some of which may influence the photocatalytic process.

The synthetic methods toward high-quality pervoskite crystals are crucial for the halide perovskite photocatalytic system. The solution-based processing technologies for perovskite films can be briefly summarized as one- and two-step methods (Burschka et al., 2013), while the most common colloidal nanocrystal synthetic methods include the hot injection and the ligand-assisted reprecipitation (LARP) (Protesescu et al., 2015; Zhang et al., 2015). Nanocrystals synthesized by the hot injection technique usually show better size distribution and higher quantum yield, but this method requests an inert atmosphere and high temperature, which is suitable for laboratorial production in small amounts. A large yield of nanocrystals could be obtained rapidly by the LARP method. However, a large amount of solvents will be generated during the processes. The yield is also limited by the ratio of polar and non-polar solvents (Ma et al., 2018). The practical use of halide perovskite nanocrystals and batch production methods have been summarized in another review from our group (Dong et al., 2018). Compared to traditional semiconductor materials (Si, Ge, GaN, and GaAs), the perovskite can be prepared in a more convenient method with low cost (Stoumpos and Kanatzidis, 2016). However, the Pb-based perovskites tend to release Pb^2+^, which is toxic to the environment (Flora et al., 2012). Although lead is allowed in well-encapsulated photovoltaic modules, it would still be preferable to find an alternative (Giustino and Snaith, 2016). Therefore, plenty of works focusing on lead-free perovskite have been reported.

Considering the above obstacles and being different from traditional photocatalytic systems, the design of the halide perovskite photocatalytic system remains challenging.

Herein, we focus on the catalyst design and reaction system design of halide perovskite materials for photocatalytic applications. First, the hydrogen evolution reaction (HER) catalyzed by perovskite photoanode/photocathode devices with p-i-n and n-i-p structures will be summarized, with focus on the unique device structures and encapsulation methods. Second, HER catalyzed by perovskite nanocrystals (NCs) and heterojunctions with schemes to improve carrier separation and photocatalytic performances will be discussed. As an important part, the CO_2_ reduction reaction catalyzed by perovskite, including the design of heterojunction and reaction system, will be summarized. Third, some special reactions that can be photocatalyzed by halide perovskite will also be summarized by centering on how to create active sites on catalyst through the design of a reaction system. At the end of the review, the current challenges and prospects for future development are suggested.

## Photoelectrochemical Anode/Cathode Devices for the Hydrogen Evolution Reaction

The electrochemical reaction occurs at the interface between the electrode and the electrolyte. For the electrochemical water splitting reaction system, the hydrogen evolution reaction (HER) occurs at the cathode surface, and the oxygen evolution reaction (OER) occurs at the anode surface. The half reactions for water splitting are as follows (Kim et al., 2020):

(1) $$\begin{array}{l} \text{HER}:2{\text{H}}_{2}\text{O}+2{\text{e}}^{-}\to {\text{H}}_{2}\left(\text{g}\right)+2\text{O}{\text{H}}^{-},\text{E}=0.00\text{V} \end{array}$$

(2) $$\begin{array}{l} \text{OER}:2\text{O}{\text{H}}^{-}\to 1/2{\text{O}}_{2}\left(\text{g}\right)+{\text{H}}_{2}\text{O}+2{\text{e}}^{-},\text{E}=1.23\text{V} \end{array}$$

The theoretical minimum voltage for electrochemical water splitting is 1.23 V. For a device constructed for water splitting, the minimal open-circuit voltage should be way higher than 1.23 V by a single cell or multiple cells in series.

### Water Splitting Driven by Perovskite Photoelectrochemical Cells

**Figure 2:**
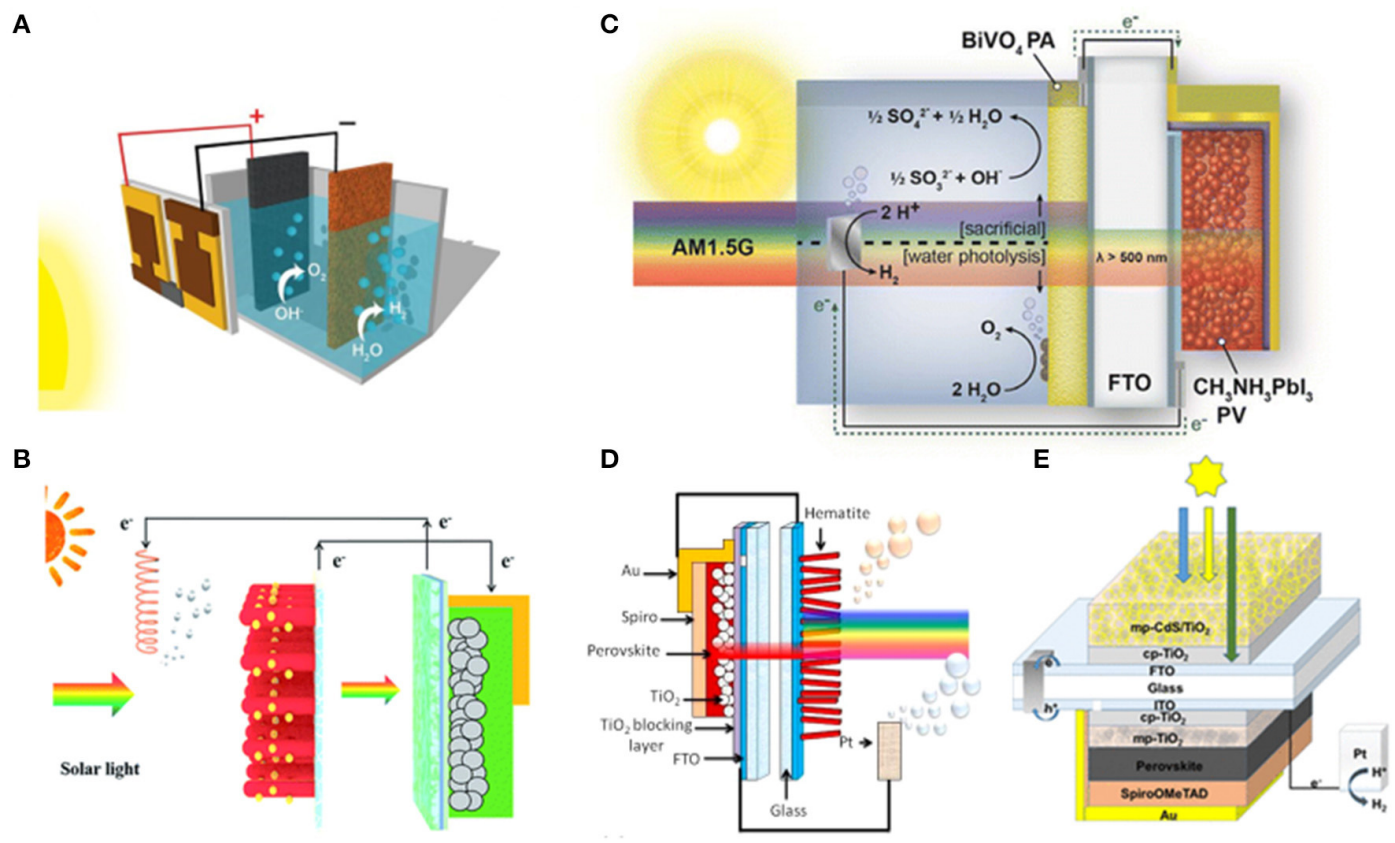
**(A)** Schematic diagram of the water-splitting device and a generalized energy schematic of the perovskite tandem cell for water splitting. Copyright © © 2014, American Association for the Advancement of Science. **(B)** Schematic diagram depicting the combination of a perovskite solar cell with a photocatalytic process. Left panel: Pt electrode. Right panel: CdS/TiO_2_ NRA photoanode and perovskite solar cells. Reproduced by permission of The Royal Society of Chemistry. **(C)** Single-junction MAPbI_3_ perovskite solar cell with the champion metal oxide photoanode material BiVO_4_ for water splitting. Copyright © © 2014, American Chemical Society. **(D)** Schematic of the dual junction perovskite solar cell/hematite photoanode tandem cell. Copyright © © 2015, American Chemical Society. **(E)** Schematic of integrated hydrogen evolution reaction (HER) device use CdS/TiO_2_ as photoanode. Copyright © © 2018, American Chemical Society.

The water splitting driven by the dye-sensitized solar cell and other solar cells have long been reported (Brillet et al., 2012; Li et al., 2015; Kang et al., 2017). In 2014, Grätzel et al. reported the first proof-of-concept water photolysis by perovskite photovoltaic device (Luo et al., 2014). The tandem perovskite solar cells assembled in series to achieve a water splitting voltage, each possessing a power conversion efficiency of 17.3% with open-circuit voltage at 1.06 V and fill factor at 0.76. A two-electrode alkaline water splitting system with NiFe-layered double hydroxide (LDH) bifunctional OER and HER catalyst electrodes is thus driven by the tandem perovskite solar cells. The obtained device shows a solar-to-hydrogen efficiency at 12.3% (Figure 2A). Li et al. reported the solar-powered photocatalysis powered by a single-junction perovskite solar cell. The photoanode is a CdS-decorated TiO_2_ nanorod array. The band gap of CdS is 2.42 eV, sensitive in the visible light region, which is narrower than TiO_2_. The energy level of the conduction band minimum (CBM) of CdS is more negative than H_2_O/H_2_, and thus favoring H_2_ generation. The CdS-decorated TiO_2_ nanorod arrays are grown on a FTO substrate, which can be directly exploited and fabricated by spin-coating or doctor-blade processing techniques. This device leads to an overall solar-to-hydrogen efficiency of 1.54% in the water splitting and a 6-fold enhancement in organic degradation rate compared with that of the CdS/TiO_2_ NRAs alone (Liu et al., 2019; Figure 2B).

The perovskite device and photoelectrode possessing different bandgaps can capture photons with various energy in the visible light spectrum, which may improve the photocatalytic activity, light absorption efficiency, and the overpotential of the reaction. Kamat et al. designed a synergistic tandem photoanode–photovoltaic device of single-junction MAPbI_3_ perovskite solar cell with the “champion” metal oxide photoanode material BiVO_4_ for water splitting. Under AM 1.5 G illumination, the BiVO_4_ absorbs the light with wavelength below 500 nm, whereas the perovskite absorbs the light in the longer wavelength range [red and near-infrared radiation (IR) region], enabling a solar-to-hydrogen conversion efficiency of 2.5% at neutral pH without external bias (Chen et al., 2015; Figure 2C). Grätzel et al. employed Mn-deposited hematite (Fe_2_O_3_) as photoanode driven by a single-junction perovskite solar cell and achieved a 2.4% solar-to-hydrogen efficiency. Since the Fe_2_O_3_ has a bandgap of 2.1 eV, the perovskite solar cell should possess a lower bandgap to absorb the long-wavelength end of the solar spectrum. An unassisted water splitting reaction can be achieved by a perovskite solar cell modified with hematite photoanode. The high-open-circuit-voltage perovskite solar cells with optical absorption up to ~800 nm can synergize with the hematite photoanode (Sabba et al., 2015; Figure 2D).

Meanwhile, the encapsulation strategy can be employed to protect the device from oxygen and moisture. Karuturi et al. use CdS/TiO_2_ as photoanode to fabricate the HER device. The photoanode is integrated on the glass side of a mixed-cation perovskite solar cell with a Pt photocathode. The encapsulated device can properly work in a mixed aqueous solution electrolyte of Na_2_S (0.25 M) and Na_2_SO_3_ (0.35 M) with a pH of ~12.5, accomplishing a 10% solar-to-hydrogen conversion efficiency. The device is encapsulated with a thin glass slide using hot-melting Surlyn, followed by edge sealing using epoxy with UV treatment (Figure 2E). Based on the methodology, various structures of perovskite photoelectrochemical cells are built by combining different encapsulation strategies with various perovskite devices and photoelectron catalysts (Karuturi et al., 2018).

### n–i–p Structure Perovskite Photoelectrochemical Cells

Sandwich-type perovskite devices are usually fabricated layer by layer on conductive glass substrate such as indium tin oxide (ITO) or fluorine-doped tin oxide (FTO). By engineering the transport layers and device structure, a flexible design in device architectures can be achieved, e.g., the n-i-p (conventional) and p-i-n (inverted) configurations (Xu et al., 2019). In the n-i-p structure, a p-type semiconductor, i.e., a hole-transporting layer, is on the top of the device, whereby the surface of the device exposed to the electrolyte will act as an oxidation catalyst, with the reduction reaction occurring on the surface electrically connected to the n side.

**Figure 3:**
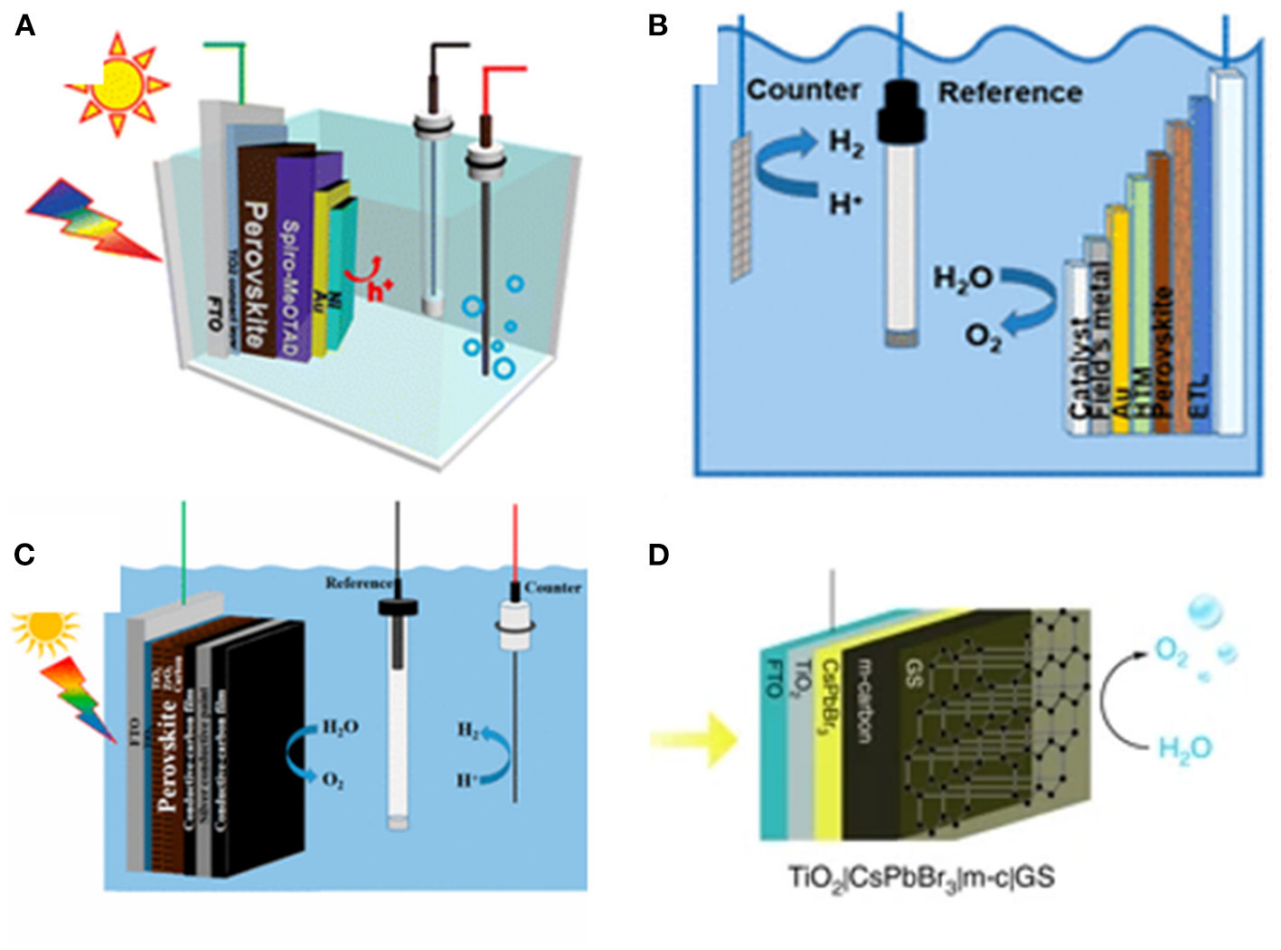
**(A)** Schematic illustration of photoelectrochemical test of a Ni-coated perovskite photoanode in a photoelectrochemical (PEC) cell using standard three-electrode system. Copyright © © 2015, American Chemical Society. **(B)** Schematic illustration of integrated photoelectrolysis cell with perovskite photoelectrode. Copyright © © 2018, American Chemical Society. **(C)** Schematic illustration of the perovskite photoanode for PEC water splitting in a standard three-electrode system. Copyright © © 2019, American Chemical Society. **(D)** Schematic illustration of the CsPbBr_3_ photoanode for PEC O_2_ evolution: TiO_2_|CsPbBr_3_|m-c|GS uses commercial conductive GS on the m-carbon layer.

Wang et al. fabricated an ultrathin bifunctional Ni top layer on perovskite photoanode, wherein the Ni layer serves as an effective physical passivation against water and a hole-transferring catalyst to enhance the photocurrent and improve the stability. The photocurrent density determined in 0.1 M Na_2_S at 0 V vs. Ag/AgCl is found to be over 10 mA/cm^2^. The photocurrent density can be maintained above 2 mA/cm^2^ for more than 15 min of continuous tests (Da et al., 2015; Figure 3A). Field's metal is defined as a low-melting-point eutectic metal. Oh et al. reported a Field's metal encapsulated perovskite device for solar water splitting. Ni film catalyst is electrodeposited on Field's metal, which also protects the underlying layer in harsh alkaline and oxidative environment. This perovskite photoelectrode can effectively operate in strong alkaline electrolyte (Nam et al., 2018; Figure 3B).

Li et al. selected the mixed-cation perovskite (5-AVA)_x_(MA)_1−x_PbI_3_ to fabricate halide perovskite photoanode of conventional electrode structure. The photovoltaic system is encapsulated with conductive carbon paste that works as a bifunctional material for effective hole extraction and collection, with silver conductive paint as a waterproof layer and hole transport. The perovskite photoanode achieves a photocurrent density of approximately 12.4 mA/cm^2^ at 1.23 V_RHE_ from the light-driven water oxidation to O_2_. The photoanode maintains over 70% of its initial current after continuous operation for 12 h (Tao et al., 2019; Figure 3C). Hintermair et al. reported the first study of light-driven water oxidation reaction in aqueous solution using all-inorganic CsPbBr_3_-based photoanodes. To prevent the CsPbBr_3_ degradation in water, the perovskite is protected by a mixture of mesoporous carbon layer and graphite sheet. The electrodes can survive in a wide pH range from 2 to 13. The champion photocurrents at a bias of 1.23 V_RHE_ can be up to 3.8 mA cm^−2^ in acidic solution (Poli et al., 2019; Figure 3D).

### p–i–n Structure Perovskite Photoelectrochemical Cells

The p-i-n structure device exposes the hole-transporting layer on the top, wherein the reduction reaction will occur on the surface of the device, while the oxidation reaction will occur on the other side.

**Figure 4:**
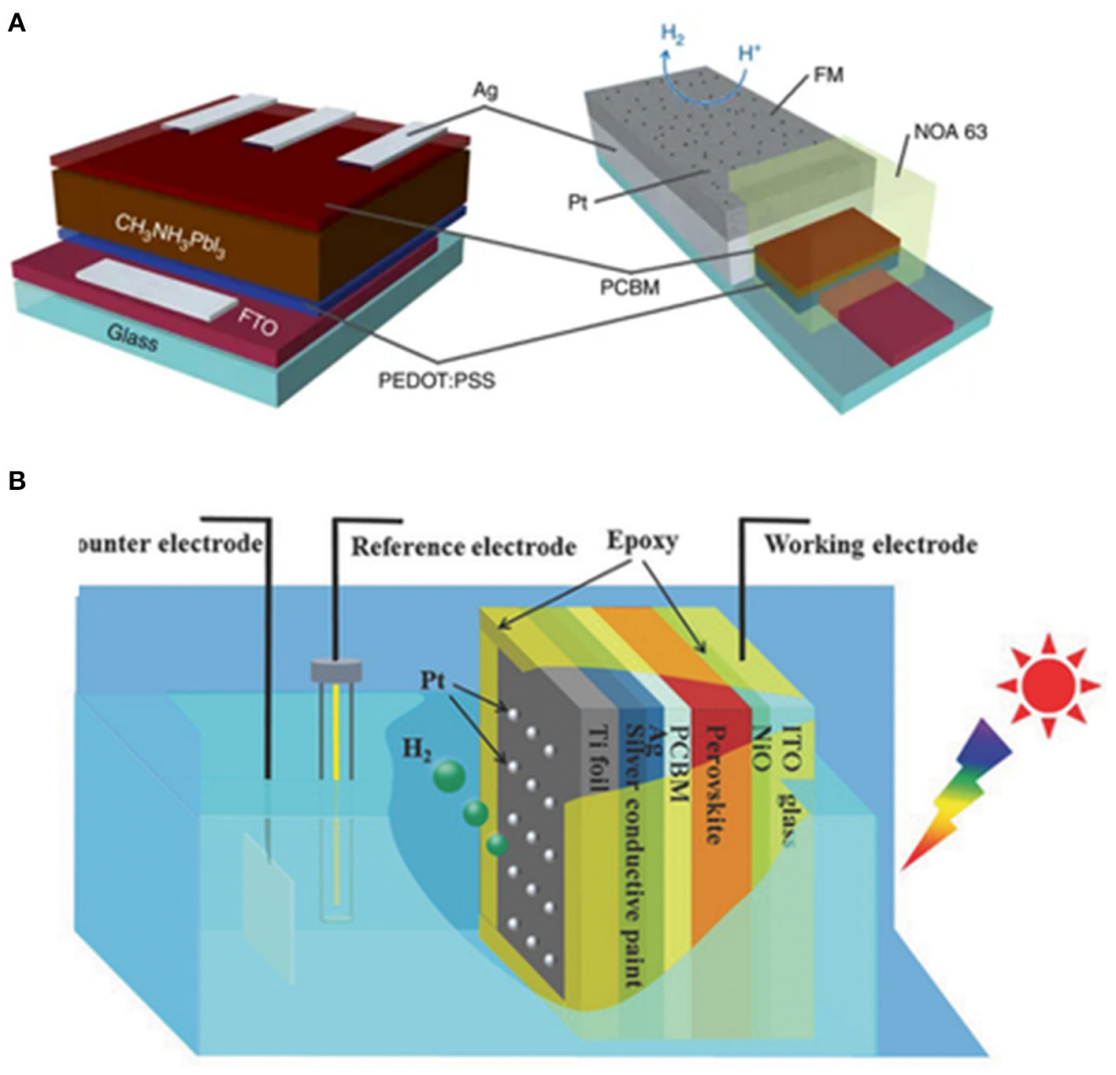
**(A)** Schematic representation of the structure of the perovskite solar cell. An inverted p-i-n configuration is used, with the general FTO/PEDOT:PSS/perovskite/PCBM/PEIE:Ag structure. **(B)** Schematic illustration of the sandwich-like MAPbI_3_ photocathode for PEC H_2_ evolution in a standard three-electrode system. © © 2018 WILEY-VCH Verlag GmbH & Co. KGaA, Weinheim.

Reisner et al. encapsulated a perovskite solar cell with a fusible InBiSn alloy, a member of Field's metal family. The InBiSn is located between the catalyst (Pt) and solar cell, serving to transport the photo-generated electrons while at the same time protect the perovskite from water. With this design, a champion photocurrent density of 9.8 mA cm^−2^ can be achieved at 0 V_RHE_. An onset potential 0.95 ± 0.03 V_RHE_ can be obtained. Under continuous illumination, the device retains over 80% of the initial photocurrent for 1 h in electrolyte solution (0.1 M borate, pH 8.5), which is a remarkable stability (Crespo-Quesada et al., 2016; Figure 4A).

Li et al. reported an MAPbI_3_-based photocathode for HER. The MAPbI_3_ photocathode delivers an onset potential at 0.95 V_RHE_ and a photocurrent density of ~18 mA cm^−2^ at 0 V_RHE_ in 0.5 M H_2_SO_4_ under AM 1.5G illumination. The reaction can last for at least 12 h under continuous illumination in water (Zhang H. et al., 2018; Figure 4B).

Martinson et al. develop an acid-compatible halide perovskite photocathode for solar-driven HER. The device is a sandwiched ITO/PEDOT:PSS/perovskite/PC_61_BM + TiO_2_ structure, wherein the poly(3,4-ethylenedioxythiophene) polystyrene sulfonate (PEDOT:PSS) is used as a hole-transport layer, perovskite Cs_0.05_(MA_0.17_FA_0.83_)_0.95_Pb(I_0.83_Br_0.17_)_3_ as a light-absorbing layer, and PC_61_BM + TiO_2_ as an electron-transport layer. Finally, a semitransparent ultrathin Pt film is deposited as a catalyst for the HER. An average photocurrent density of 10.5 cm^−2^ at 0 V_RHE_ and photovoltage of 0.68 V are obtained. The device also exhibits remarkable stability in the acidic H_2_SO_4_ electrolyte (Kim et al., 2019).

To prevent the erosion of water to the perovskite, Zou et al. designed a perovskite photocathode encapsulated by bulk material-based fusible In–Bi–Sn alloy with Pt as active catalyst. Silver, as a layer to improve conductivity, is deposited between the passivation layer and the electron-transporting layer (ZnO). The device shows a 1.37-V open-circuit voltage and a 2.50-mA/cm^2^ short-circuit current density. The total power conversion efficiency is 1.91%. The faradaic efficiency of the CsPbBr_3_-based photocathode for the HER is calculated to be over 90% (Gao et al., 2018).

Cu(In, Ga)(S, Se)_2_ (CIGS) is a well-studied material for high-performance photovoltaic technology with a record efficiency of 22.9% (Kato et al., 2018). Recently, Nam et al. reported a CIGS photocathode with ZnS/CdS double buffer layer. This photocathode shows the champion photocurrent density at 35.5 mA/cm^2^ at 0 V_RHE_ among those reported works on CIGS. The device shows an onset potential of 0.66 V_RHE_. Combined with IrO_*x*_ anode on a lead halide perovskite solar cell, a remarkable solar-to-hydrogen efficiency of 9.04% and stability for over 6.5 h can be achieved (Koo et al., 2020). Ahmad et al. designed a Cs-FA-MA triple-cation-based perovskite photocathode with Al-doped ZnO protective layer for HER. The device can work continually for 18 h before complete failure with a starting photocurrent at 14.3 mA cm^−2^ (Ahmad et al., 2019).

### Summary of Designing Principle

Based on the above discussion, in order to design a state-of-the-art high-performance perovskite photoelectronic device, the following principles should be preferred: (1) the device must be highly efficient in light absorption and charge transport to ensure the abundancy in charge supply; (2) the device has to be stable in the operation medium or protected by encapsulation, but with the functional catalyst exposed to the electrolyte; (3) the photoelectrode and the electrolyte must be compatible through a proper design in device structure by considering the redox potential of the reactant. Depending on the specific reaction and different design strategies, both the n-i-p and p-i-n photoelectrochemical devices may have the potential to be high-performance devices.

Further efforts have been made in the following aspects: (I) Catalyst structure design, such as the use of nanoarray structures to improve light absorption and carrier transportation (Sabba et al., 2015; Liu et al., 2019); (II) Device architecture engineering such as the device encapsulation and surface active sites modification (Nam et al., 2018; Ahmad et al., 2019; Poli et al., 2019; Tao et al., 2019); (III) Developing new perovskite material with better stability and performance (Karuturi et al., 2018; Ahmad et al., 2019; Kim et al., 2019; Liu et al., 2019; Tao et al., 2019); (IV) New compatible catalytic material development such as NiFe or BiVO_4_ (Luo et al., 2014; Chen et al., 2015); and (V) New device fabricating method such as ALD (Gao et al., 2018). All of these explorations deserve further and insightful investigations. The materials and reaction systems reported in literatures are summarized in Table 1.

**Table 1:** A brief summary of the materials and reaction systems involved in perovskite-based photoelectronic device.

**Perovskite**	**Application**	**Electrolyte**	**Photocathode**	**Photoanode**	**Light source**	**Open-circuit voltage**	**Photocurrent density**	**Solar-to-hydrogen efficiency**	**References**
Organometal trihalide perovskite	HER, Organics degradation	NaOH, Na_2_SO_4_	–	CdS/TiO_2_	AM 1.5G	1.05 V	1.15 mA cm^−2^	1.54%	Liu et al., 2019
MAPbI_3_	HER	Na_2_SO_3_(phosphate)	–	BiVO_4_	AM 1.5G	1.3 V	3.7 mA cm^−2^	2.50%	Chen et al., 2015
MAPbI_3_	Water splitting	1 M NaOH (pH=13.6)	–	Fe_2_O_3_ (Mn doped Fe_2_O_3_)	AM 1.5G	About 1 V	3.5 mA cm^−2^	2.40%	Sabba et al., 2015
Cs_0.05_(MA_0.17_ FA_0.83_)_0.95_Pb(I_0.83_Br_0.17_)_3_	HER	Na_2_S, Na_2_SO_3_	–	CdS/TiO_2_	AM1.5G	1.095 V	7.8 mA cm^−2^	>10%	Karuturi et al., 2018
MAPbI_3_	Solar-to-fuel	Na_2_S	–	MAPbI_3_-based photoanode with an ultrathin Ni surface layer	AM 1.5G	0.95 V	10 mA cm^−2^	–	Da et al., 2015
MAPbI_3_	Water splitting	K-Borate, KOH	–	–	AM 1.5G	0.98 V	21 mA cm^−2^	–	Nam et al., 2018
(5-AVA)_x_(MA)_1−x_PbI_3_	Water splitting	KOH	–	Conductive-carbon	AM 1.5G	1.23 V	12.4 mA cm^−2^	–	Tao et al., 2019
CsPbBr_3_	OER	KOH	–	Graphite-protected CsPbBr_3_ perovskite	AM 1.5G	1.3 V	>2 mA cm^−2^	82.3% (O_2_)	Poli et al., 2019
MAPbI_3_	Water splitting	NaOH	NiFe LDH	–	AM 1.5G	1.06 V	10 mA cm^−2^	12.3%	Luo et al., 2014
MAPbI_3_	HER	Borate	MANH_3_PbI_3_ (InBiSn)	–	AM 1.5G	1.0 ± 0.09 V	9.8 mA cm^−2^	–	Crespo-Quesada et al., 2016
MAPbI_3_	HER	H_2_SO_4_	MANH_3_PbI_3_	–	AM 1.5G	–	18 mA cm^−2^	–	Zhang H. et al., 2018
Cs_0.05_(MA_0.17_ FA_0.83_)_0.95_ Pb(I_0.83_Br_0.17_)_3_	HER	H_2_SO_4_	PEDTO: PSS	PC61BM/TiO_2_/Pt	0.5 Sun illumination	–	>10 mA cm^−2^	–	Kim et al., 2019
CsPbBr_3_	HER	Na_2_HPO_4_/ NaH_2_PO_4_	CsPbBr_3_	–	AM 1.5G	–	1.2 mA cm^−2^	0.64%	Gao et al., 2018
Cs_0.05_(MA_0.17_ FA_0.83_)_0.95_ Pb(I_0.83_Br_0.17_)_3_	Water splitting	K_2_HPO_4_, KH_2_PO_4_, and H_2_SO_4_	NiO_x_/ perovskite/PCBM /AZO/FM/Pt NPs	NiO_x_/perovskite/ PCBM/AZO/FM/Pt NPs	AM 1.5G	0.97 V	14.3 mA cm^−2^	–	Ahmad et al., 2019

## Perovskite-Based Photocatalytic System Toward the Hydrogen Evolution Reaction

Instead of fabricating complicated devices, HER can be achieved directly by heterogeneous photocatalysts. The HER driven by pure perovskite-based catalysts and heterojunction-type perovskite-based catalysts will be discussed at this part.

### Hydrogen Evolution Reaction by Heterogeneous Perovskite-Based Catalysts

**Figure 5:**
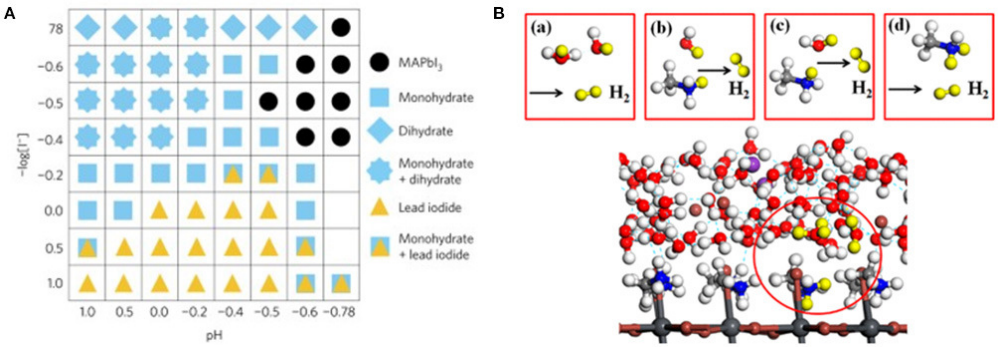
**(A)** Constructed phase map as a function of [I^−^] and [H^+^]. Each symbol indicates the stable precipitate phases in saturated solutions at each [I^−^] and [H^+^] concentration. Copyright © © 2018, American Chemical Society. **(B)** Four possible reaction pathways for photocatalytic H_2_ generation on the MAPbI_3_ surface. Copyright © © 2018, American Chemical Society.

The work reported by Park et al. (2017) of splitting hydrogen iodide (HI) into H_2_ and ${\text{I}}_{3}^{-}$ with MAPbI_3_ under solar irradiation is considered as the milestone for the photocatalytic application of halide perovskite. In this work, Park et al. stabilized the MAPbI_3_ in aqueous HI saturated solution by forming a dynamic equilibrium state, whereby the dissolution of MAPbI_3_ reaches an equilibrium with the precipitation of MA^+^ and ${\text{PbI}}_{3}^{-}$ ions. The authors discovered that MAPbI_3_ can exist in different phases depending on I^−^ and H^+^ concentration, among which the MAPbI_3_ phase is only stable under specific conditions (Figure 5A). Later, Wang et al. investigate the mechanistic role of H^+^ in MA^+^ during the photocatalytic HER reaction through the theoretical first-principle calculation (Wang et al., 2018a). They discovered that an intermediate Pb–H is generated by proton released from an MA^+^ ion. The intermediate subsequently reacted with a proton released from another MA^+^ ion to generate H_2_. The intermediate state MA^+^ ion would replace the lost H from the solution via the Grotthuss mechanism (Figure 5B). As a follow-up work, they made another first-principle calculation by replacing the Pb with Sn, I with Br, and MA^+^ cation with other organic cations, including the FA^+^ cation. As a result, they predicted that FAPbI_3_ exhibits the best HER performance with a 10-fold reaction rate compared to MAPbI_3_ (Wang L. et al., 2018).

Although the perovskite family are of good light-absorbing materials, achieving the effective carrier separation and building the catalytically active sites on the surface are the key factors to construct a highly efficient photocatalytic system. A proper design in catalyst nanostructure or heterojunction structure is crucial and challenging.

**Figure 6:**
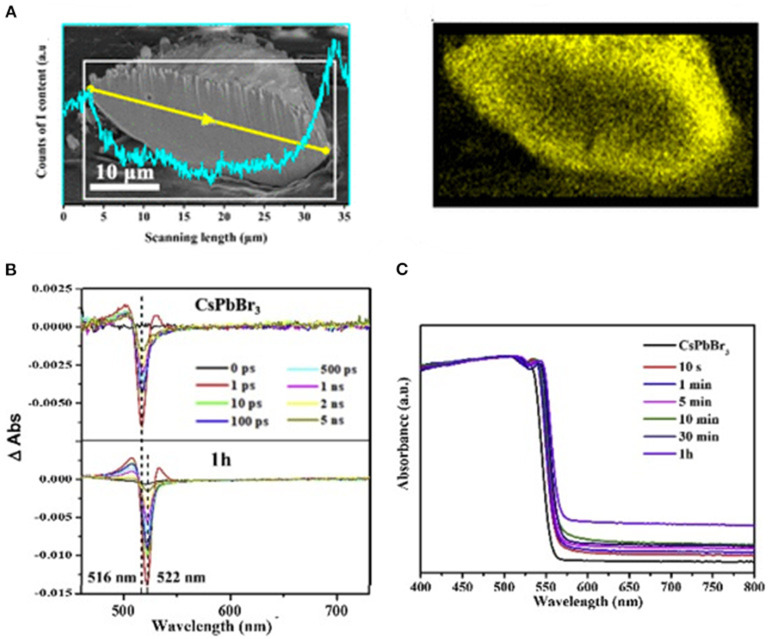
**(A)** An SEM image of the cross section of a bisected MAPbBr_3−x_I_*x*_ particle. The profile presented in cyan shows the variation of the I-content determined by EDX scanning along the yellow line profile and in the region marked with a white rectangle. Copyright © © 2018, American Chemical Society. **(B)** Transient absorption spectra of CsPbBr_3_ before and after the 1 h of the ion-exchange reaction. © © 2019 Elsevier B.V. All rights reserved. **(C)** The UV-vis diffuse reflectance spectra of CsPbBr_3−x_I_x_ powders taken at different halide exchange reaction times. © © 2019 Elsevier B.V. All rights reserved.

Wu et al. designed a mixed halide perovskite MAPbBr_3−x_I_*x*_ prepared by halide-exchange method. The iodide and bromine ions inside perovskite show a gradient distribution. The unique elemental profile builds up a funnel band structure from the surface to the core (Figure 6A) and, hence, improves the carrier transport from the interior to the surface. By depositing Pt on the surface, the photocatalyst exhibits a photocatalytic activity for the HER at a rate of 651.2 μmol h^−1^ and solar-to-chemical conversion efficiency of 1.05% under visible light (100 mW cm^−2^, λ ≥420 nm) (Wu et al., 2018b). The same group discovered that a similar bandgap funnel structure could also be applied to the all-inorganic perovskite CsPbBr_3−x_I_x_, whereas the CsPbBr_3_ samples can hardly be efficient for the HER even when loaded with Pt as cocatalyst (Guan et al., 2019; Figures 6B,C). Zhao et al. also employed MAPb(I_1−x_Br_*x*_)_3_ (*x* = 0–0.20) as HER photocatalyst. Different from the intentional design of the gradient distribution in Wu's work, they prepared the perovskite with isotropic elemental distribution, which can be stabilized in mixed HBr/HI solution. The highest HER activity (1,471 μmol h^−1^g^−1^) is obtained by MAPb(I_1−x_Br_*x*_)_3_ (*x* = 0.10) under visible light. The MAPb(I_1−x_Br_*x*_)_3_ (*x* = 0.10) shows a 40-time higher activity than that of pure MAPbI_3_ (Zhao et al., 2019d).

Gu et al. investigated the lead-free MA_3_Bi_2_I_9_ perovskite for photocatalytic HER. Similar to the lead perovskite, MA_3_Bi_2_I_9_ also shows phase stability in HI solution. Upon the loading of Pt as a cocatalyst on the surface, the MA_3_Bi_2_I_9_ shows a 14-time improvement in photocatalytic activity up to 169.21 μmol g^−1^ h^−1^, which implies the potential of lead-free perovskite for photocatalysis (Guo Y. et al., 2019).

### HER Catalyzed by Heterojunction-Structure Perovskite Catalysts

**Figure 7:**
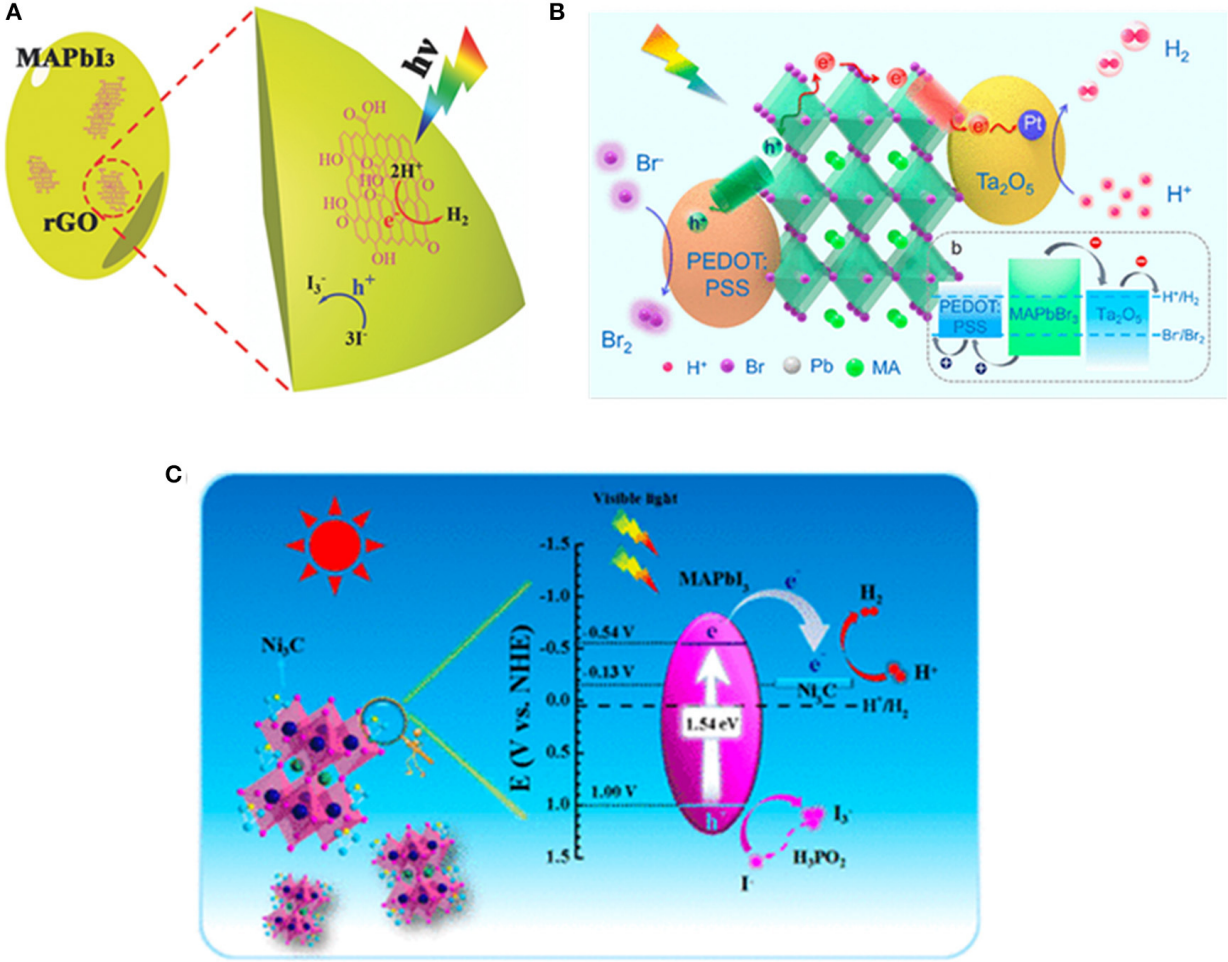
**(A)** Schematic illustration of the HER using MAPbI_3_/rGO. © © 2018 WILEY-VCH Verlag GmbH & Co. KGaA, Weinheim. **(B)** Schematic illustration of the reaction mechanism for MAPbBr_3_ with Pt/Ta_2_O5 and PEDOT:PSS as the electron- and hole-transporting materials, respectively. Copyright © © 2019, American Chemical Society. **(C)** Schematic band diagram of Ni_3_C/MAPbI_3_ for the HI-splitting photocatalytic reaction. Copyright © © 2019, American Chemical Society.

For the optoelectronic device fabricated with bulk or thin-film materials, heterojunction is usually employed to accelerate the separation and transport of charge carriers. For nanoscale photocatalytic system, heterojunction structure is also seen as an effective strategy to enhance the charge transport. Wu et al. reported a MAPbI_3_/rGO composite and demonstrated that the composite powder is highly efficient for the HER with high stability. The composite is prepared by dispersing GO powders in aqueous HI acid solution saturated with MAPbI_3_, followed by exposure to visible light (λ ≥420 nm), whereby the GO is reduced to rGO, and hence, the MAPbI_3_/rGO composite is obtained (Figure 7A). The Pb–O–C bridges the perovskite and rGO, and enables the injection of electrons into the rGO, whereas the photo-generated holes can oxidize I^−^ to ${\text{I}}_{3}^{-}$ (Wu et al., 2018c). Wang et al. showed that MAPbBr_3_ NCs can be stabilized in aqueous HBr solution similar to the MAPbI_3_ in HI solution (Park et al., 2017). By hybridizing MAPbBr_3_ with PEDOT:PSS and Pt/Ta_2_O_5_ nanoparticles as hole- and electron-transport materials, the rate of photocatalytic HER is boosted to 52 times (Wang H. et al., 2018; Figure 7B).

Zhao et al. decorated the MAPbI_3_ with Ni_3_C for the HER with the rate up to 2,362 μmol g^−1^ h^−1^, way higher than with Pt/MAPbI_3_ (534 μmol g^−1^ h^−1^) and pristine MAPbI_3_ (43 μmol g^−1^ h^−1^) (Figure 7C). The enhancement of Ni_3_C/MAPbI_3_ is triggered by the well-aligned Fermi level and inherent electrical conductivity (Zhao et al., 2019c).

Li et al. demonstrated that few-layer black phosphorus (BP) could effectively serve as an electron trapper, which transports electrons from MAPbI_3_ at the interface of BP/MAPbI_3_. The photocatalytic HER rate reaches 3,742 μmol h^−1^ g^−1^ under visible light (Li et al., 2019).

Wang et al. compared the photocatalytic HER activities of Pt/MAPbI_3_ and Pt/TiO_2_-MAPbI_3_ in HI-water solution. They discovered that by introducing the electron-transporting layer material TiO_2_ into the catalyst, the activity will be greatly improved. Compared to Pt/MAPbI_3_, the Pt/TiO_2_-MAPbI_3_ system shows an 89-time higher activity in photocatalytic HER (Wang et al., 2018b). Xiao et al. employed the CsPbBr_3_ QDs/Pt-TiO_2_ for the HER in a gas/solid photocatalytic system. The CsPbBr_3_ QDs/Pt–TiO_2_ composite powder is scraped onto a glass plate located above a mixed solution of methanol and water in a customized reactor. The powder can catalyze the reaction of water vapor with methanol to produce H_2_. However, compared with the HER catalyzed by the organic–inorganic hybrid perovskite in liquid phase, there is much room for the further improvement in activity (Xiao et al., 2019).

The heterojunction-type photocatalytic systems based on the halide perovskite are summarized in Table 2.

**Table 2:** Summary of heterojunction-type halide perovskite-based photocatalyst.

**Catalyst**	**Reduction/ oxidation material (R:/O:)**	**Type**	**Solute**	**Light source**	**HER rate**	**Solar-to-chemical conversion efficiency**	**Apparent quantum efficiency**	**References**
MAPbI_3_/Pt	R:Pt O:MAPbI_3_	Schottky junction	HI	Λ ≥ 475 nm	57 μmol·g^−1^·h^−1^	0.81%	–	Park et al., 2017
CsPbBr_3−x_I_x_/Pt	R:Pt O:CsPbBr_3−x_I_x_	Schottky junction	HBr	120 mW·cm^−2^, λ ≥ 420 nm	1,120 μmol·g^−1^·h^−1^		2.15%	Guan et al., 2019
MA_3_Bi_2_I_9_/Pt	R:Pt O:MA_3_Bi_2_I_9_	Schottky junction	HI	300 W Xe-lamp, 400 nm cut-off filter	169.21 μmol·g^−1^·h^−1^	0.48%	–	Guo Y. et al., 2019
MAPbBr_3−x_I_x_/Pt	R:Pt O:MAPbBr_3−x_I_x_	Schottky junction	HBr/HI	300 W Xe-lamp, 420 nm cut-off filter	2,604.8 μmol·g^−1^·h^−1^	1.06%	–	Wu et al., 2018b
MAPb(I_1−x_Br_x_)_3_	–	–	HI/HBr	300 mW cm^−2^ Xe-lamp, λ ≥ 420 nm	1,471 μmol·g^−1^·h^−1^	1.42%	–	Zhao et al., 2019d
MAPbI_3_/rGO	R:rGO O:MAPbI_3_	Schottky junction	HI	300 W Xe-lamp, 420 nm cut-off filter	938.9 μmol·g^−1^·h^−1^	–	1.4%(λ = 450 nm)	Wu et al., 2018c
Pt/TiO_2_-MAPbI_3_	R:Pt/TiO_2_ O:MAPbI_3_	Type II	HI	300 W Xe-lamp, 420 nm cut-off filter, λ > 420 nm	7,633 μmol·g^−1^·h^−1^	0.86%	Λ = 420 nm, 70%	Wang et al., 2018b
Pt/Ta_2_O_5_-MAPbBr_3_-PEDOT:PSS	R:Pt/Ta_2_O_5_ O:PEDOT:PSS	Type I	HBr	λ > 420 nm, 150 mW cm^−2^	96.3 μmol·h^−1^	–	Λ = 420 nm, 16.4%	Wang H. et al., 2018
Ni_3_C/MAPbI_3_	R:Ni_3_C O:MAPbI_3_	Schottky junction	HI	300 mW·cm^−2^ Xe-lamp, 420 nm cut-off filter, λ ≥ 420 nm	2,362 μmol·h^−1^	0.91%	Λ = 420 nm, 16.6%	Zhao et al., 2019c
CsPbBr_3_ QDs/Pt-TiO_2_	R:Pt-TiO_2_ O:CsPbBr_3_	Type II	water and methanol	300 W Xe-lamp, λ > 420 nm	About 130 μmol·g^−1^	–	–	Xiao et al., 2019
BP/MAPbI_3_	R:BP O:BP	Type I	HI	300 mW·cm^−2^ Xe-lamp, λ ≥ 420 nm	3,742 μmol·g^−1^·h^−1^	0.93%	Λ = 420 nm, 23.2%	Li et al., 2019

## Perovskite-Based Catalytic System for the Photo-Reduction of CO_2_

The CO_2_ reduction has been considered as a promising strategy to reduce the global warming and convert the wasted CO_2_ gas into useful chemicals, especially when converted by environmentally friendly non-fossil energy source such as electrocatalysis, photoelectrocatalysis, and photocatalysis. However, CO_2_ is an extremely stable molecule. The bonding energy of C–O can be as high as 750 kJ/mol, much higher than C–H (430 kJ/mol) and C–C (336 kJ/mol). Depending on the number of carbon atoms in one molecule, the product can be generally classified into C1 products like carbon monoxide (CO), formic acid (HCOOH), methanol (CH_3_OH), formaldehyde (HCHO), methane (CH_4_), or C2 products such as ethylene (CH_2_CH_2_), ethanol (C_2_H_5_OH), and acetate (CH_3_COOH) (Zhu and Li, 2019). Different products are favored at different redox potentials. The standard reduction potentials of different products from CO_2_ are summarized in Table 3.

**Table 3:** Summary of the standard reduction potentials of different products of CO_2_ reduction reaction in H_2_O (25°C, 1.0 atm).

**Electrochemical CO_**2**_ reduction reaction**	**Standard potentials (V_**SHE**_)**
CO_2_ (g) + 4H^+^ + 4e^−^ = C(s) + 2H_2_O (l)	0.210
CO_2_ (g) + 2H_2_O (l) + 4e^−^= C(s) + 4OH^−^	−0.627
CO_2_ (g) + 2H^+^ + 2e^−^= HCOOH (l)	−0.250
CO_2_ (g) + 2H_2_O (l) + 2e^−^ = HCOO^−^(l) + OH^−^	−1.078
CO_2_ (g) + 2H^+^ + 2e^−^ = CO (g) + H_2_O (l)	−0.106
CO_2_ (g) + 2H_2_O (l) + 2e^−^ = CO (g) + OH^−^	−0.934
CO_2_ (g) + 4H^+^ + 4e^−^ = CH_2_O (l) + H_2_O (l)	−0.070
CO_2_ (g) + 2H_2_O (l) + 4e^−^ = CH_2_O (l) + 4OH^−^	−0.898
CO_2_ (g) + 6H^+^ + 6e^−^ = CH_3_OH (l) + H_2_O (l)	0.016
CO_2_ (g) + 5H_2_O (l) + 6e^−^ = CH_3_OH (l) + 6OH^−^	−0.812
CO_2_ (g) + 8H^+^ + 8e^−^ = CH_4_ (g) + H_2_O (l)	0.169
CO_2_ (g) + 6H_2_O (l) + 8e^−^ = CH_4_ (g) + 8OH^−^	−0.659
2CO_2_ (g) + 2H^+^ + 2e^−^ = H_2_C_2_O_4_ (aq)	−0.500
2CO_2_ (g) + 2e^−^ = C_2_${\text{O}}_{4}^{2-}$ (aq)	−0.590
2CO_2_ (g) + 12H^+^ + 12e^−^ = CH_2_CH_2_ (g) + 4H_2_O (l)	0.064
2CO_2_ (g) + 8H_2_O (l) + 12e^−^ = CH_2_CH_2_(g) + 12OH^−^	−0.764
2CO_2_ (g) + 12H^+^ + 12e^−^ = CH_3_CH_2_OH (l) + 3H_2_O (l)	0.084
2CO_2_ (g) + 9H_2_O (l) + 12e^−^ = CH_3_CH_2_OH (l) + 12OH^−^	−0.744

Although the perovskite-based photoelectrochemical cells for the HER has been investigated as previously discussed, no literature on the photoelectrochemical reduction of CO_2_ has been reported as far as we know, which nonetheless is worthwhile for future investigations. From the photocatalytic or photoelectrocatalytic point of view, it is noteworthy that the halide perovskite possesses a suitable band structure for the CO_2_ reduction. In recent years, some studies on the CO_2_ reduction with halide perovskite driven by visible light have been reported (Bera and Pradhan, 2020; Shyamal and Pradhan, 2020; Wang H. et al., 2020). For the reported work involving the CO_2_ reduction using perovskite-based photocatalysts, the detailed reaction conditions have been summarized and listed in Table 4. The reported studies suggest that perovskites can play as candidates for photocatalytic CO_2_ reduction. Different from previous reviews, this part will be centered on the design of catalyst structure and reaction system for the photocatalytic CO_2_ reduction reaction, with detailed discussion in: (1) efficient absorption and light management, (2) surface/interface engineering, (3) stability improvement, (4) product selectivity, and (5) design of reaction system.

**Table 4:** Summary of the photocatalytic CO_2_ reduction system and performances by perovskite-based photocatalysts.

**Catalyst**	**Light source**	**Reaction atmosphere**	**Reduction/ oxidation material (R:/O:)**	**System**	**Reduction/oxidation products**	**Selectivity**	**Electron consumption rate**	**References**
CsPbBr_3_ QDs CsPbBr_3_ QDs/GO	AM 1.5 G 100 W Xe-lamp	Ethyl acetate (liquid)	R: GO	Type II	CO, CH_4_, H_2_	>99%	29.8 μmol·g^−1^·h^−1−^	Xu et al., 2017
CsPbBr_3_ QDs	AM 1.5 G 300 W Xe-lamp	Ethyl acetate/H_2_O (liquid)	R: CsPbBr_3_	Single-component	CO, CH_4_, H_2_	>99%	20.9 μmol·g^−1^·h^−1^	Hou et al., 2017
Cs_2_AgBiBr_6_	AM 1.5 G 100 W Xe-lamp	Ethyl acetate (liquid)	R: Cs_2_AgBiBr_6_ O: Cs_2_AgBiBr_6_	Single-component	CO, CH_4_	100%	17 μmol·g^−1^·h^−1^	Zhou et al., 2018
CsPbBr_3_ NC/Pd NS	150 W Xe-lamp	CO_2_ and H_2_O vapor (gas)	R: Pd O: CsPbBr_3_	Type II	CO, CH_4_, H_2_	93.5%	33.79 μmol·g^−1^·h^−1^	Xu et al., 2018b
CsPbBr_3_ QDs/g-C_3_N_4_	300 W Xe-lamp	Acetonitrile/H_2_O (liquid)	R: g-C_3_N_4_ O: CsPbBr_3_	Type II	CO, CH_4_	-	CO: 149 μmol·g^−1^·h^−1^	Ou et al., 2018
CsPbBr_3_ NC/a-TiO_2_	AM 1.5 G 150 W Xe-lamp	Ethyl acetate/Isopropanol (100:1,v:v) (liquid)	R: a-TiO_2_	Type II	CO, CH_4_, H_2_	95.5%	64.45 μmol·g^−1^·h^−1^	Xu et al., 2018a
CsPbBr_3_ QDs@ZIF-67	AM 1.5 G 100 W Xe-lamp	CO_2_ and H_2_O vapor (gas)	R: ZIF-67 O: CsPbBr_3_	Type II	CH_4_, CO (O_2_)	100%	29.63 μmol·g^−1^·h^−1^	Kong et al., 2018
CsPb(Br_0.5_/Cl_0.5_)_3_	AM 1.5 G 300 W Xe-lamp	Ethyl acetate (liquid)	–	Single-component	CO, CH_4_	99%	CO+CH_4_: 266.4 μmol·g^−1^·h^−1^	Guo S.-H. et al., 2019
CsPbBr_3_ QDs/UiO-66(NH_2_)	300 W Xe-lamp	Ethyl acetate/H_2_O (liquid)	R: UiO-66(NH_2_) O: CsPbBr_3_ + UiO-66(NH_2_)	Type II	CO, CH_4_ (O_2_)	100%	18.5 μmol·g^−1^·h^−1^	Wan et al., 2019
MAPbI_3_ QDs/Fe-MOF	300 W Xe-lamp	Ethyl acetate/H_2_O (liquid)	R:Fe-MOF	-	CO, CH_4_ (O_2_)	100%	112 μmol·g^−1^·h^−1^	Wu et al., 2019
CsPbBr_3_ NC/BZNW/MRGO	150 W Xe-lamp	CO_2_ and H_2_O vapor (gas)	R: MRGO O: CsPbBr_3_	Type II	CO, CH_4_	CO_2_: 100% CH4: 96.7%	52.02 μmol·g^−1^·h^−1^	Jiang et al., 2019
Co(II) doped CsPbBr_3_/Cs_4_PbBr_6_	300 W Xe-lamp	Pure water (liquid)	R: Co(II) O: CsPbBr_3_	Type II	CO, CH_4_ (O_2_)	100%	CO:11 μmol·g^−1^·h^−1^	Mu et al., 2019
CsPbBr_3_-Re(CO)_3_Br(dcbpy)	AM 1.5 G 150 W Xe-lamp	Toluene/isopropanol (9:1,v:v) (liquid)	R: Re(CO)_3_Br(dcbpy) O: CsPbBr_3_	Type II	CO, H_2_	95%	73.34 μmol·g^−1^·h^−1^	Kong et al., 2020
CsPbBr_3_ NCs/Mxene	300 W Xe-lamp	Ethyl acetate (liquid)	R: Mxene	Type II	CO, CH_4_	100%	110.64 μmol·g^−1^·h^−1^	Pan et al., 2019
CsPbBr_3_@TiO-CN	300 W Xe-lamp	Ethyl acetate/H_2_O (200:1 v:v) (liquid)	R: TiO-CN	Type II	CO, CH_4_	100%	CO: 12.9 μmol·g^−1^·h^−1^	Guo X.-X. et al., 2019
Fe(II) doped CsPbBr_3_	450 W Xe-lamp	Ethyl acetate/H_2_O (liquid)	Blank	Single-component	CO, CH_4_	–	CH_4_: 6.1 μmol·g^−1^·h^−1^ CO: 3.2 μmol·g^−1^·h^−1^	Shyamal et al., 2019
CsPbBr_3_-Ni(tpy)	300 W Xe-lamp	Ethyl acetate/Water (49:1, v:v) (liquid)	R: Ni(tpy)	Type II	CO, CH_4_	–	1,252 μmol·g^−1^·h^−1^	Chen et al., 2020
α-Fe_2_O_3_/Amine-RGO/CsPbBr_3_	AM 1.5 G	CO_2_ and H_2_O vapor (gas)	R: CsPbBr_3_ O: α-Fe_2_O_3_	Z-scheme	CH_4_, CO, H_2_ (O_2_)	CH4: 93.4%	80.95 μmol·g^−1^·h^−1^	Jiang et al., 2020
Mn-doped CsPb(Br/Cl)_3_	300 W Xe-lamp	Ethyl acetate (liquid)	–	Single-component	CO, CH_4_	–	CO: 213 μmol·g^−1^·h^−1^ CH_4_: 9 μmol·g^−1^·h^−1^	Liu et al., 2020
Co_X%_@CsPbBr_3_/Cs_4_PbBr_6_	300 W Xe-lamp	Acetonitrile/water/methanol (liquid)	R: Co(II) O: CsPbBr_3_/Cs_4_PbBr_6_	Type II	CO	–	CO: 122 μmol·g^−1^·h^−1^	Dong et al., 2020a
MAPbI_3_@PCN-221(Fe)	300 W Xe-lamp	Ethyl Acetate/water (liquid)	R: PCN-221(Fe) O: MAPbI_3_	–	CO, CH_4_	–	CO and CH_4_: 1,559 μmol g^−1^	Wu et al., 2019

### Efficient Absorption and Light Management

**Figure 8:**
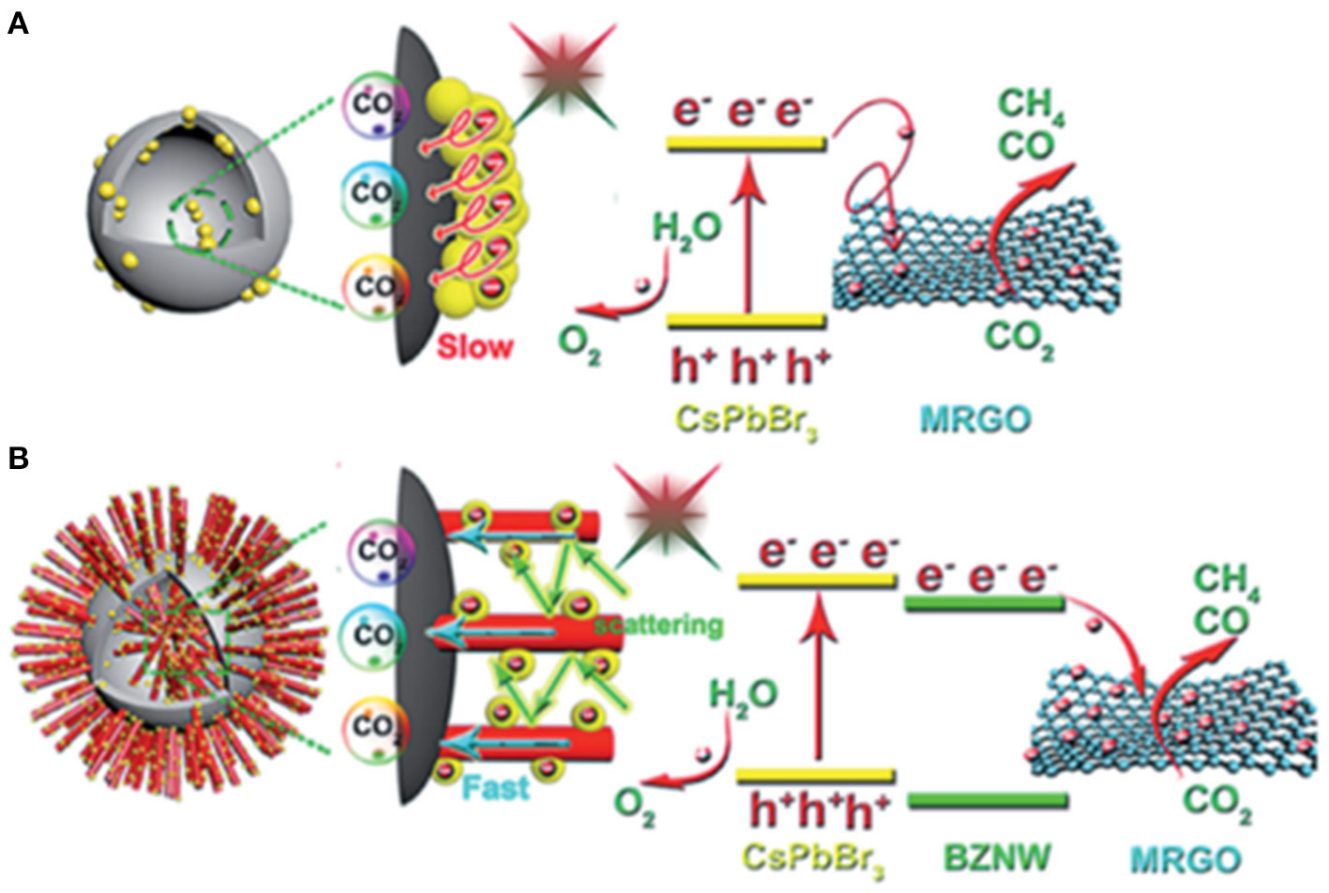
Schematic illustration of the energy band structure and possible transfer process of photo-induced carriers: **(A)** CsPbBr_3_ NC/MRGO and **(B)** CsPbBr_3_ NC/BZNW/MRGO hybrids under visible light irradiation. Reproduced by permission of The Royal Society of Chemistry.

As previously mentioned, light absorption and management are crucial for the photocatalysis. To achieve efficient light absorption, materials with smaller band gaps are usually preferred. However, lowering the conduction band minimum will reduce the energy of the photo-generated electrons. A rational design in catalyst structure, e.g., the catalyst morphology, could greatly improve the light absorption efficiency. Kuang et al. synthesized a novel ternary hybrid catalyst with CsPbBr_3_ NC, branched ZnO nanowire (BZNW), and macroporous graphene scaffold (MRGO) through a multistep methodology (Jiang et al., 2019; Figure 8). Meanwhile, the scattering among the ZnO nanowires can further improve the light absorption similar to the light management in solar cell technology. The CH_4_ selectivity of the composite ternary heterojunction-type catalyst can reach as high as 96.7% compared with the 78.2% obtained from the pure CsPbBr_3_ NCs.

In a heterojunction structure, the perovskite serves as light absorber, with cocatalyst enhancing the carrier transport and providing active sites. A proper engineering on the perovskite and cocatalyst can be beneficial in improving the light absorption and carrier physics. Metal organic framework (MOF) has attracted widespread attention due to the wide range of light absorption, large specific surface area, and adjustable structure (Dhakshinamoorthy et al., 2016). Zhong et al. developed a surface-functionalization strategy for CsPbBr_3_ QDs and obtained CsPbBr_3_ QDs/UiO-66(NH_2_) nanocomposite catalyst via an ultrasonic method (Wan et al., 2019). The amino group on the UiO-66(NH_2_) MOF enables a tight bonding through the chemical interaction between perovskite and MOF. By modulating the perovskite/UiO-66(NH_2_) ratio, the light absorption efficiency can be properly optimized. For the time being, it is not yet established which light management strategy will generate the optimized performance, which deserves future efforts for research.

### Surface/Interface Engineering

The modulation of the carrier dynamics at the catalyst surface/interface, e.g., suppressing the carrier recombination and improving the carrier transport, is one of the main challenges to design a photocatalyst. Various efforts have been dedicated to promoting the effective separation and transport of photo generated carriers for perovskite-based catalysts, including ionic doping, surface functionalization, and heterojunction nano-structuring. Huge amount of studies has demonstrated the effectiveness in improvement of carrier transport promoted by fabricating heterojunction structure in terms of engineering of band structure and good contact at interface.

**Figure 9:**
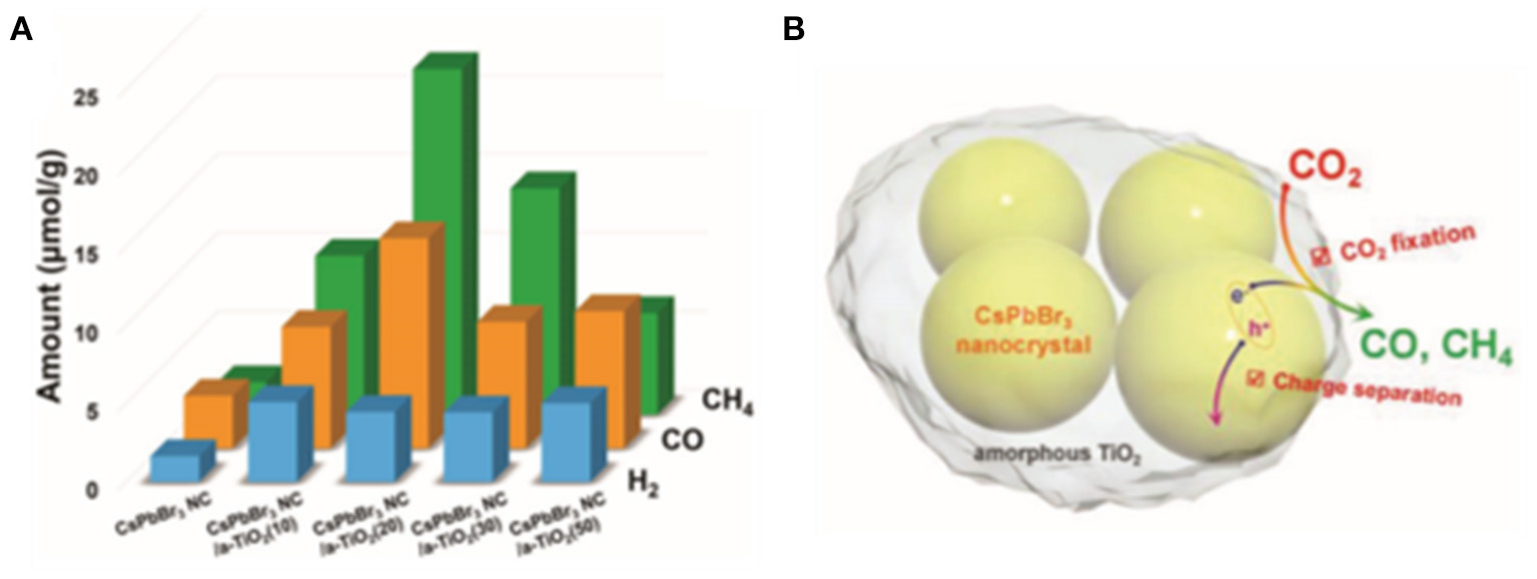
**(A)** Photocatalytic CO_2_ reduction test results. **(B)** A schematic illustration on the enhanced charge separation and CO_2_ fixation in a TiO_2_-encapsulated CsPbBr_3_ NCs. © 2018 WILEY-VCH Verlag GmbH & Co. KGaA, Weinheim.

Kuang et al. designed the amorphous TiO_2_-encapsulated CsPbBr_3_ nanocrystalline composite catalyst by modulating the volume of tetrabutyl titanate precursors via liquid-phase synthesis (Xu et al., 2018a). TiO_2_ is a well-accepted wide band-gap semiconductor material with excellent chemical stability. The encapsulation effectively inhibits radiative recombination and promotes carrier separation, and hence improves the catalytic activity (Figure 9).

Zhang et al. functionalized the graphitic carbon nitride surface with CsPbBr_3_ NCs decorated with titanium-oxide species. The design effectively promotes the charge separation and introduces the metal catalytic active sites in the catalytic system (Guo X.-X. et al., 2019). Verified by the high-resolution XPS, the two components are tightly bound through the chemical interaction between tBr-N and Pb-O. A similar strategy is also employed to construct the CsPbBr_3_/MXene composite catalyst and other photocatalytic systems. Ti_3_C_2_T_x_, commonly called as MXene material with excellent conductivity, adjustable interlayer, and hydrophilic surface, can serve as efficient charge transport medium and supporting material (Zhang Y. et al., 2018). The CsPbBr_3_/MXene composite catalyst is successfully prepared by *in situ* growth method and utilized as photocatalyst for the CO_2_ reduction in ethyl acetate. The MXene is found to be effective in promoting the transfer of photogenerated electrons from CsPbBr_3_ (Pan et al., 2019).

**Figure 10:**
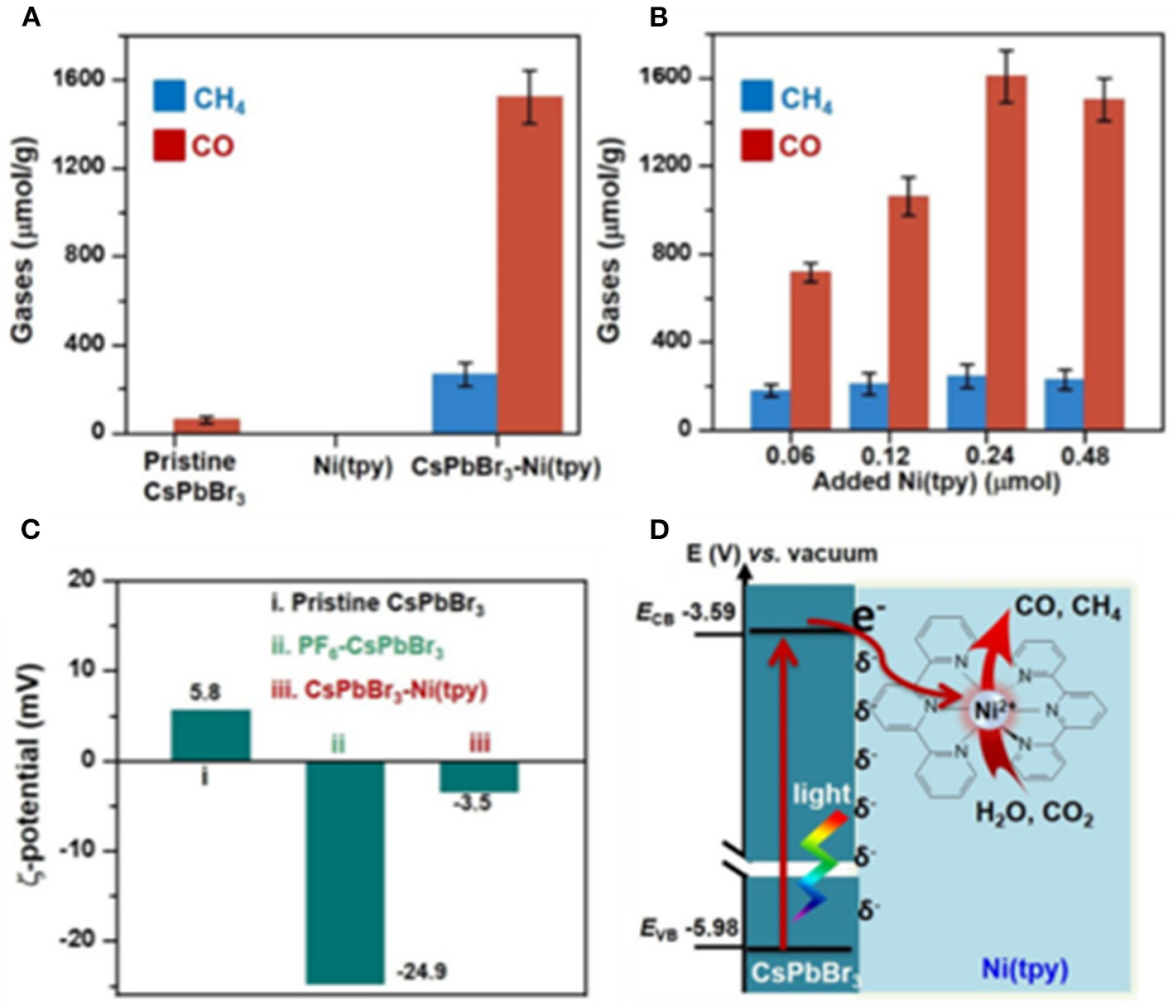
**(A)** Production of gases using various photocatalysts based on the pristine CsPbBr_3_, the pristine Ni(tpy), and CsPbBr_3_-Ni(tpy). **(B)** Changes in gas production as a function of the added Ni(tpy); **(C)** variations in the zeta (ζ) potentials of the pristine CsPbBr_3_, PF_6_-CsPbBr_3_, and CsPbBr_3_-Ni(tpy) in EA. **(D)** Schematic representation of the CsPbBr_3_-Ni(tpy) photocatalyst system. © © 2018, American Chemical Society.

A CsPbBr_3_-Ni(tpy) catalyst is reported by Li et al., wherein the PF_6_-modified CsPbBr_3_ NCs can bind strongly to Ni(tpy) through a strong electrostatic interaction. According to their investigation, coupling metal complexes with perovskite NCs shows the following advantages: (1) the abundant ionic sites on perovskite NCs can be readily modified for immobilizing metal complexes; (2) the immobilized metal complexes can serve as catalytic centers, which are expected to enhance the catalytic performance of the CO_2_ reduction; and (3) the metal complexes can serve as the electron sinks, which are able to accept multiple photo-excited electrons from the perovskite NCs in a stepwise manner (Chen et al., 2020). This is particularly important for the multi-electron reduction of CO_2_, which can proceed at relatively low overpotentials. The polypyridyl rings in Ni(tpy) can extract and store electrons, whereas the Ni-center serves as the catalytic active site as well as electron sink to suppress the charge recombination. The CsPbBr_3_-Ni(tpy) is found to show a 26-time enhancement in activity compared with that of the pure CsPbBr_3_ NCs, indicating that an immobilizing metal complex on CsPbBr_3_ can possibly be an effective strategy for the design of an efficient CO_2_ reduction photocatalytic system (Figure 10).

The engineering of the surface and interface nano-structure to promote charge separation and transport is of great significance for their catalytic performance. In short, in-depth studies on the precise synthesis, band structure engineering, interface nanostructure characterizations, and reaction pathway are needed to understand the functions of all components, which allows the design of high-performance halide perovskite-based photocatalytic system.

### Stability Improvement

**Figure 11:**
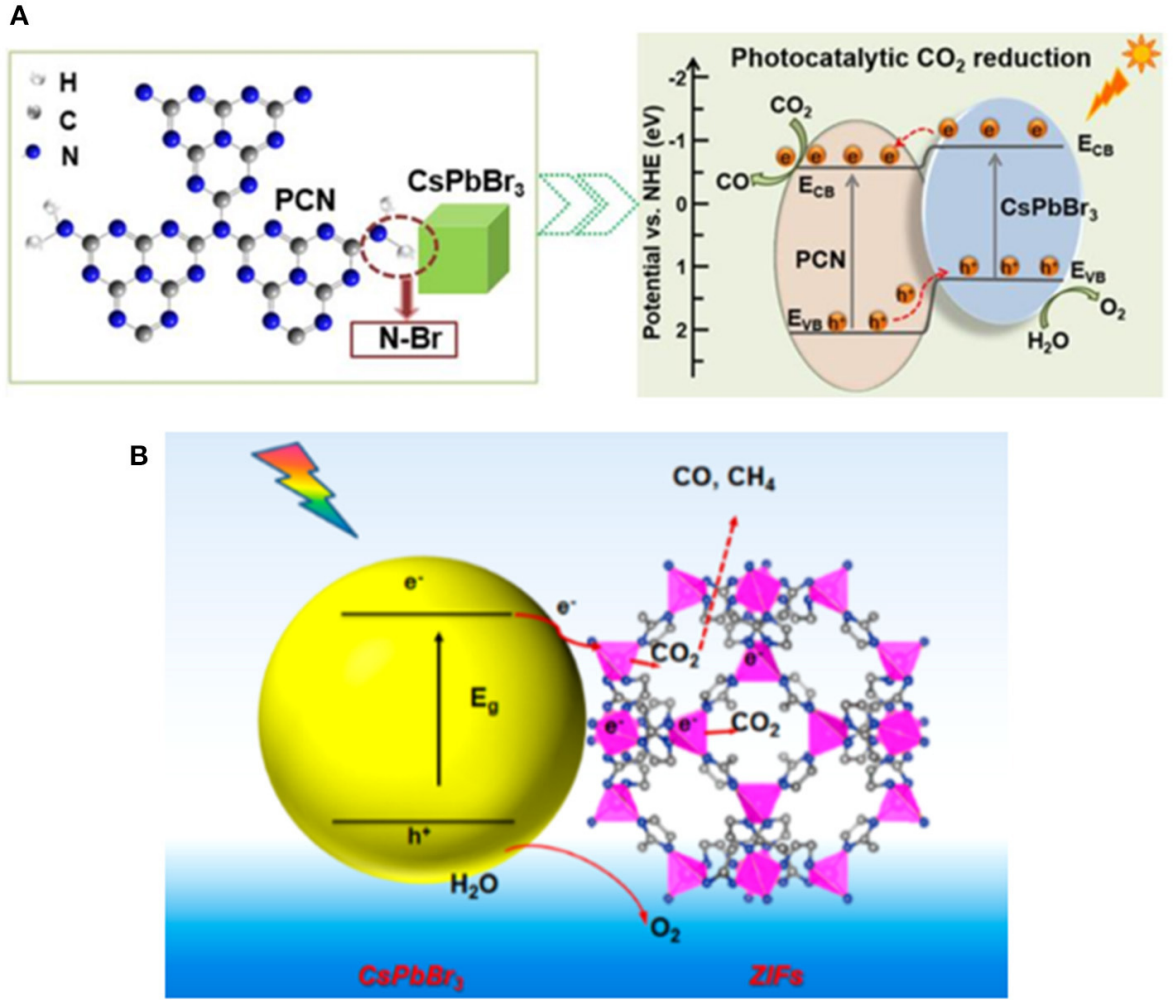
**(A)** Left: Schematic representation of amino-assisted anchoring of CsPbBr_3_ perovskite QDs on porous g-C_3_N_4_ via N-Br chemical bonding; Right: Band structures of the composite photocatalyst. © © 2018 Wiley-VCH Verlag GmbH & Co. KGaA, Weinheim. **(B)** Schematic illustration of CO_2_ photoreduction process of CsPbBr_3_/ZIFs. Copyright © © 2018, American Chemical Society.

Halide perovskite is an ionic crystal with low formation energy, which may devastate their thermal stability, light stability, and moisture stability. It is commonly considered that the stability issue may limit their applications compared to the traditional semiconducting materials (Wang et al., 2016). Despite their intrinsic limitation in stability, reasonable surface engineering strategies can be employed to address the stability issue. It is considered that the QDs passivated or wrapped by a solid material can be more chemically stable in photoreaction. Zhong et al. constructed a CsPbBr_3_ QDs/g-C_3_N_4_ composite photocatalyst with satisfactory activity and stability (Ou et al., 2018). The porous g-C_3_N_4_ with amine-rich surface and CsPbBr_3_ QDs form a contact via the N–Br bond (Figure 11A). The product rate up to 148.9 μmol·g^−1^·h^−1^ demonstrates the effectiveness of the unique structure between the porous g-C_3_N_4_ and CsPbBr_3_ QDs on the photocatalytic CO_2_ reduction. The interaction between g-C_3_N_4_ and the perovskite can also improve the stability through the passivation effect on surface structure. The stability tests suggest that after three cycles, activity losses lower than 10.3 and 2.4% can be achieved in acetonitrile/water and ethyl acetate/water, respectively.

Similar to the CsPbBr_3_-TiO_2_ core-shell structure (Xu et al., 2018a), Chen et al. successfully prepared the CsPbBr_3_@ZIF core-shell catalyst by *in situ* synthetic strategy, as shown in Figure 11B (Kong et al., 2018). The hydrophobicity of ZIF surface structure decreases the intrusion of water and hence improve the stability of CsPbBr_3_ QDs. In this scenario, Co ion in the ZIF structure serves as a catalytically active center. It has also been reported that Co-doped CsPbBr_3_/Cs_4_PbBr_6_ can be prepared in hydrophobic hexafluorobutyl methacrylate (Mu et al., 2019). It is clarified that Co-doping can transport photo generated electrons to the surface of CsPbBr_3_/Cs_4_PbBr_6_ NCs. The above work has suggested that the well-designed core-shell structure can effectively protect the perovskite from the moisture in the environment and hence stabilize the perovskite.

### Product Selectivity

For the reported results, it can be found that the main products from the photocatalytic CO_2_ reduction include CO and CH_4_, with formic acid as the only senior product reported in literature (Dong et al., 2020a). CO and CH_4_ are considered as the two important raw chemicals for C1 chemistry. It has been demonstrated that a proper design in photocatalytic structure has a significant effect on selectivity of the CO_2_ reduction. There is hardly any systematic study on the product selectivity for the perovskite-based photocatalytic CO_2_ reduction by far.

**Figure 12:**
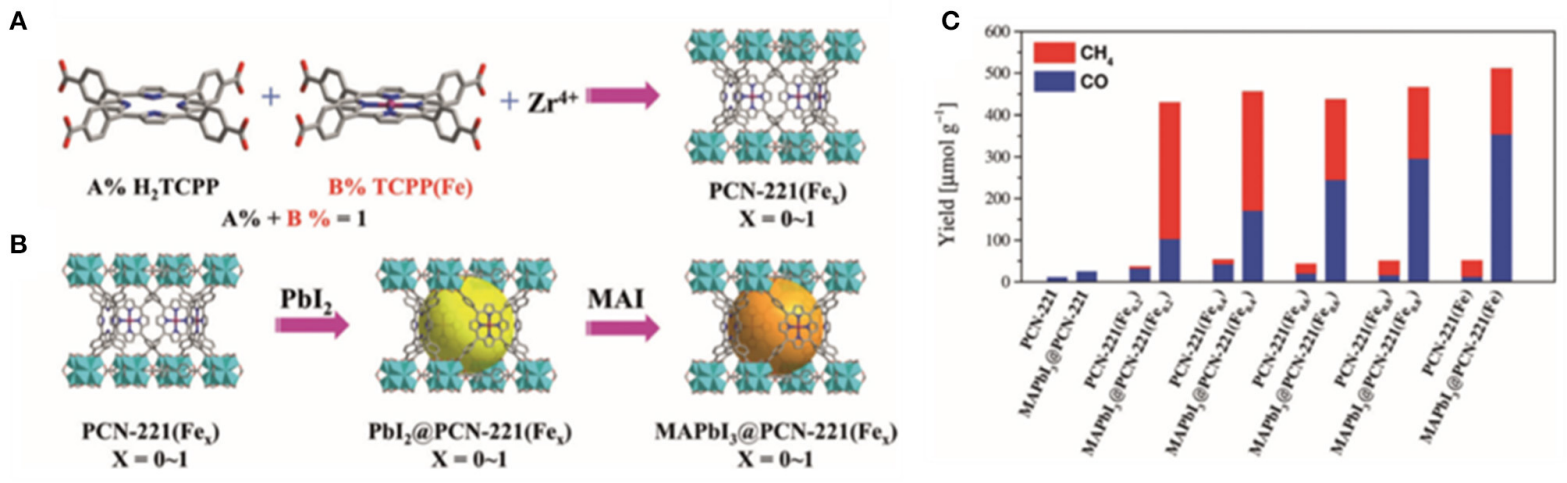
Schematic illustrations for the synthesis of **(A)** PCN-221(Fe_x_) and **(B)** MAPbI_3_ QDs (large spheres) encapsulated in the pores of PCN-221(Fe_x_) by a sequential deposition route (MAI = CH_3_NH_3_I). **(C)** The yields for CO_2_ reduction to CH_4_ and CO with PCN221(Fe_x_) and MAPbI_3_@PCN-221(Fe_x_) as photocatalysts in the CO_2_ saturated ethyl acetate/water solution after 25 h of irradiation. © 2019 Wiley-VCH Verlag GmbH & Co. KGaA, Weinheim.

Lu et al. encapsulated MAPbI_3_ QDs with iron-based MOFs through a continuous deposition method, and successfully prepared PCN-221(Fe_x_) and MAPbI_3_@PCN-221(Fe_x_) catalysts with various Fe contents (Wu et al., 2019; Figure 12). Their results indicate that the Fe content can strongly affect the selectivity. For the MOFs without Fe, CO is found to be the main reduction product, whereas for the catalyst PCN-221(Fe_x_), the selectivity to CH_4_ product increased along with the activity improvement when increasing the Fe content. Besides, the H_2_ and liquid products are not detected from the reaction system. Kuang et al. suggested that the CO product may partially be derived from the photo-oxidation of ethyl acetate (Xu et al., 2017). However, Zhang et al. believed that CO is mainly derived from the CO_2_ molecule, and ethyl acetate is not the carbon source of CO. In-depth investigation and reliable characterizations on the reaction pathway and mechanism is needed to clarify the dispute.

**Figure 13:**
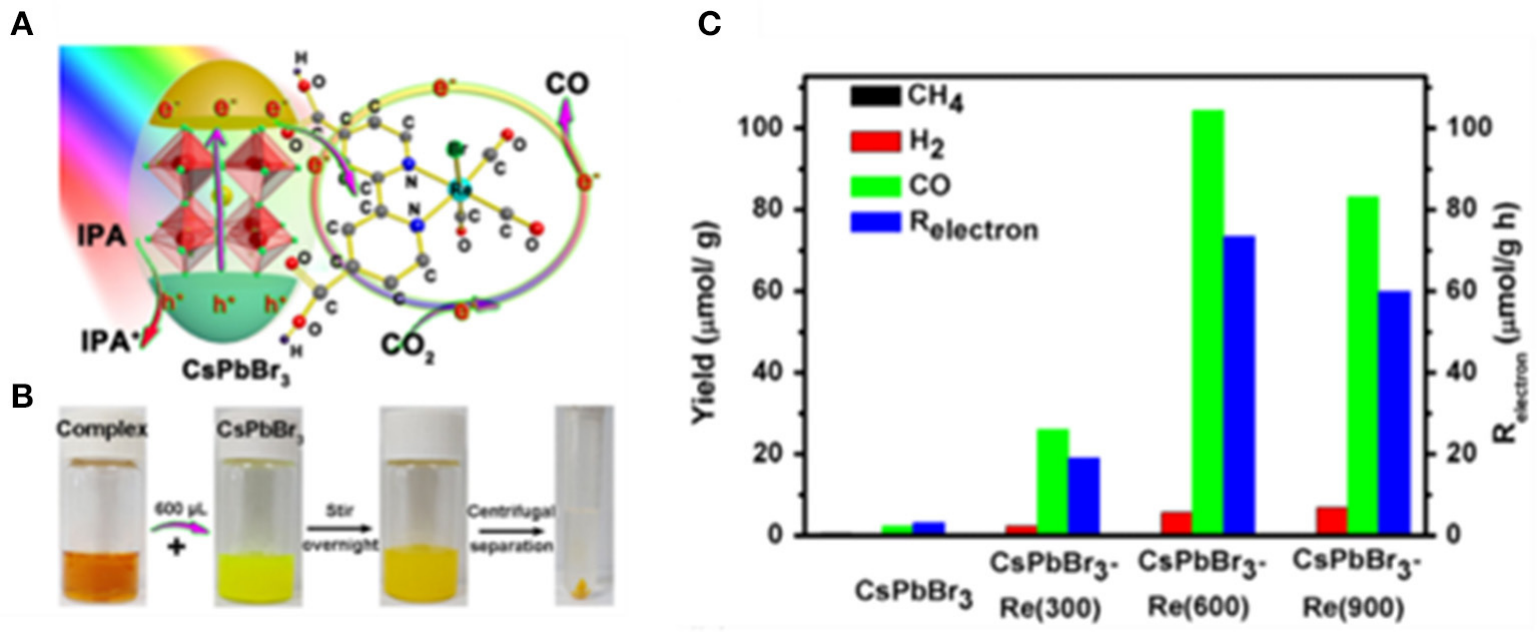
**(A)** Schematic diagram of the working principle of CsPbBr_3_-Re(CO)_3_Br(dcbpy) composite in CO_2_ reduction. **(B)** Photographs of samples during the synthetic process of CsPbBr_3_-Re(600) composite. **(C)** Photocatalytic CO_2_ reduction performances: Product yield after 3 h of reaction. © © 2019 WILEY-VCH Verlag GmbH & Co. KGaA, Weinheim.

The tricarbonyl Re(I) complexes are regarded as highly selective homogeneous catalysts to convert CO_2_ to CO, which can easily be deactivated due to the instability. This problem can be solved by anchoring complex molecules on the semiconductor surface (Kuehnel et al., 2017). Chen et al. reported CsPbBr_3_-Re(CO)_3_Br(dcbpy) heterogeneous photocatalyst by immobilizing metalorganic complex molecules on the CsPbBr_3_ NCs surface (Kong et al., 2020; Figure 13). The reaction is carried out in toluene with isopropanol as the electron donor. It shows that Re(CO)_3_Br(dcbpy) act as a cocatalyst and improves the transport of photo-generated electrons from CsPbBr_3_ to complex molecules. The CO selectivity reached 95% with optimized conditions.

As previously mentioned, Kuang et al. synthesized a novel CsPbBr_3_ NC/BZNW/MRGO ternary hybrid catalysis by multistep methodology NCs. Compared to the 78.2% CH_4_ selectivity of CsPbBr_3_ NCs in this work, the CH_4_ selectivity of the composite ternary heterojunction catalyst can be up to 96.7% (Jiang et al., 2019).

It can be inferred that the selectivity could be difficult to modulate when the reaction is occurring on the surface of perovskite. A properly designed heterojunction structure may transfer electrons from perovskite to the cocatalyst with better activity and selectivity.

### Design of Reaction System

**Figure 14:**
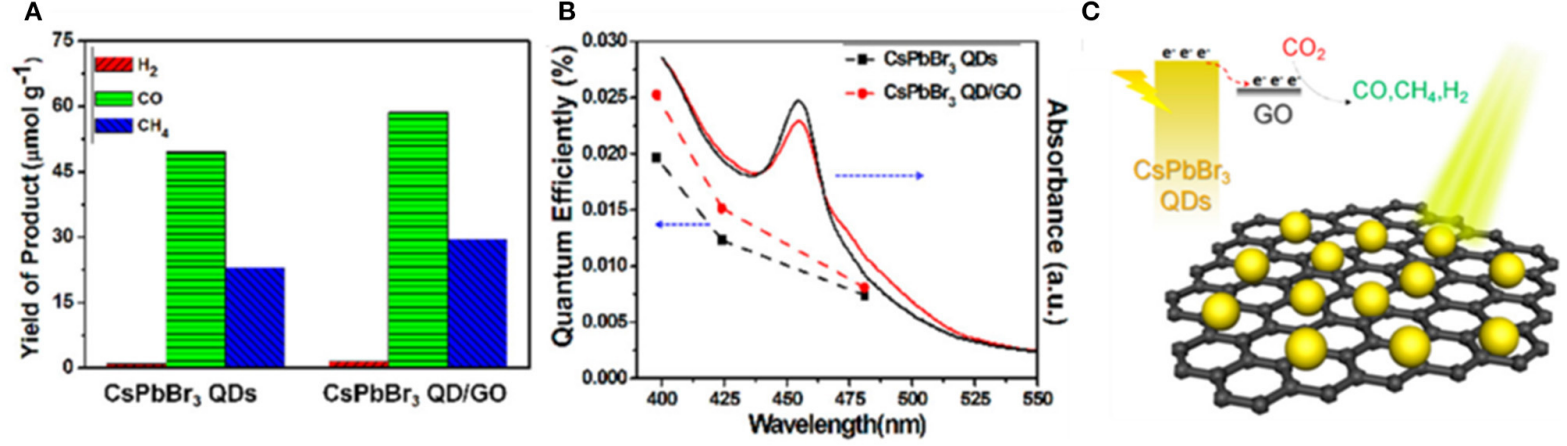
**(A)** Photocatalytic performance: yield of the CO_2_ reduction products after 12 h of photochemical reaction. **(B)** UV–vis absorption spectra and the external quantum efficiency plots. **(C)** Schematic diagram of CO_2_ photoreduction over the CsPbBr_3_ QDs/GO photocatalyst. Copyright © © 2017, American Chemical Society.

Most studies on photocatalytic CO_2_ reduction so far have selected ethyl acetate as a reaction solvent to protect the perovskite catalysts from degradation in the polar solvent. Kuang et al. designed a CsPbBr_3_/graphene oxide heterojunction structure to achieve effective carrier separation. This is the first report of CO_2_ reduction catalyzed by halide perovskite-based materials (Xu et al., 2017). The ethyl acetate is chosen as the solvent for its high solubility for CO_2_ and mild polarity to protect perovskite from degradation. Moreover, their experimental results show that a non-polar solvent is beneficial to inhibit the competing HER and also improves the selectivity of photocatalytic CO_2_ reduction (Figure 14). They suggest that the carbon of the product CO comes mainly from CO_2_ and partially from ethyl acetate.

Kuang et al. utilizes the lead-free perovskite Cs_2_AgBiBr_6_ for the CO_2_ reduction (Zhou et al., 2018). Due to the intrinsic instability of the perovskite, they found that the polar solvent (such as the dimethyl formamide and acetone) will dissolve the perovskite. However, the protonic solvent (isobutanol) will promote the ligand exchange and lead to degradation of the perovskite. For comparison, low-polarity solvents (such as the ethyl acetate, chloroform, etc.) and non-polar solvents (octane) will maintain a perovskite lattice structure. Although the ligands on the surface can reduce surface defects to protect the catalyst, this insulating layer will also slow down the charge transport on the catalyst.

**Figure 15:**
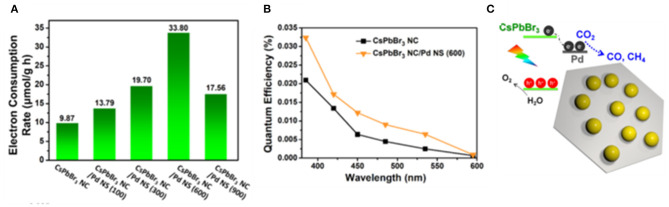
Photocatalytic CO_2_ reduction performances: **(A)** electron consumption rates under visible light illumination (>420 nm). **(B)** Quantum efficiency plots as a function of various wavelengths measured under monochromatic illumination. **(C)** Sketch of the composite material. Copyright © © 2018, American Chemical Society.

Instead of the solid–liquid reaction, the solid–gas reaction could be a solution to avoid the accumulation of moisture on the perovskite. Kuang et al. constructed a zero-dimensional CsPbBr_3_ NCs/two-dimensional Pd nanosheet catalysts with various ratios (Xu et al., 2018b; Figure 15). In this work, the Pd is employed as a cocatalyst. Carriers are effectively separated and transferred through a Schottky contact formed at the interface. Meanwhile, the Pd surface act as the catalytic active sites to boost the molecular conversion rate. Isotope experiments show that CO and CH_4_ originated from CO_2_. The results show that the optimized catalyst achieved the highest electron consumption rate and reaction rate at 33.79 μmol·g^−1^·h^−1^.

As summarized in Table 4, the polarity should be the primary factor for consideration when selecting the solvent in perovskite catalysis. Employing different solvents or reaction medium may lead to totally different reaction pathways. In addition, the interaction between surface ligands and solvent would greatly affect the perovskite structure and stability.

Kuang et al. successfully anchored the CsPbBr_3_ NCs on α-Fe_2_O_3_ single crystal nanorods with amine-functionalized rGO nanosheets. The α-Fe_2_O_3_/Amine-rGO/CsPbBr_3_ catalyst is used for visible light-driven photocatalytic CO_2_ reduction (Jiang et al., 2020). In a full photocatalytic CO_2_ reduction reaction, oxidation reaction occurs on the α-Fe_2_O_3_, whereas the reduction reaction occurs on CsPbBr_3_. the electrons generated in α-Fe_2_O_3_ annihilate with the holes generated in CsPbBr_3_ by using the amine-rGO as a bridge. The equation of a gas–solid photocatalytic reaction is:

(3) $$\begin{array}{l} \text{C}{\text{O}}_{2}\;\left(\text{g}\right)+2{\text{H}}_{2}\text{O }\left(\text{g}\right)=\text{C}{\text{H}}_{4}\;\left(\text{g}\right)+2{\text{O}}_{2}\;\left(\text{g}\right) \end{array}$$

This work is a proof-of-concept demonstration for the novel design and regulation of a Z-scheme photocatalytic structure based on halide perovskite. Optimizing the catalytic performance by increasing the loading or the visible light absorption of NCs deserve further investigation.

## Perovskite for Organic Chemical Degradation

Studies centering on advanced oxidation processes (AOPs) for the degradation of synthetic organic species have been wildly reported. The AOP relies on the generation of highly reactive radical species mainly as OH by using solar, chemical, or other forms of excitation, which allows the destruction of a wide range of organic chemical substrate with no selectivity (Gaya and Abdullah, 2008).

A variety of strategies have been developed to improve the photocatalytic AOP efficiency. The summary of the photocatalytic AOPs employing the perovskite-based catalysts are listed in Table 5.

**Table 5:** The summery of perovskite-based organic chemical photoelectrochemical degradation.

**Photocatalyst**	**Light source**	**Substrate**	**Active species**	**Degradation**	**References**
MASnI_3_/TiO_2_ (1:9)	300 W Xe-lamp	Rhodamine B	${\text{O}}_{2}^{-}$ > e^−^ > h^+^, OH	97%, 40 min	Zhang et al., 2017
CsPbX_3_ QDs	Visible light	Methyl orange	${\text{O}}_{2}^{-}$ > h^+^,·OH	CsPbCl_3_: 90%, 100 min CsPbBr_3_: 89%, 100 min	Gao et al., 2017
CsPbBr_3_ QDs	100 mW·cm^−2^ UV filter	2-Mercaptobenzothiazole	–	100%, 100 min	Cardenas-Morcoso et al., 2019
CsPb(Br_1−x_Cl_x_)_3_-Au	300 W Xe-lamp	Sudan Red III	–	71%, 360 min	Feng et al., 2019
Cs_2_AgBiBr_6_	λ > 420 nm	Rhodamine B	${\text{·O}}_{2}^{-}$	~98%, 120 min	Zhang et al., 2019
7%-Ag-CsPbBr_3_/CN	300 W Xe-lamp, UV filter	7-Aminocephalosporanic acid	h^+^, OH > e^−^,${\text{·O}}_{2}^{-}$	~92.79%, 140 min	Zhao et al., 2019b
CsPbX_3_/CN	300 W Xe lamp, 420 nm filter	6-Aminopenicillanic acid	h^+^, OH > e^−^, ${\text{O}}_{2}^{-}$	CsPbCl_3_/CN: 78.11%, 120 min CsPbBr_3_/CN: 83.31%, 120 min	Zhao et al., 2019a

**Figure 16:**
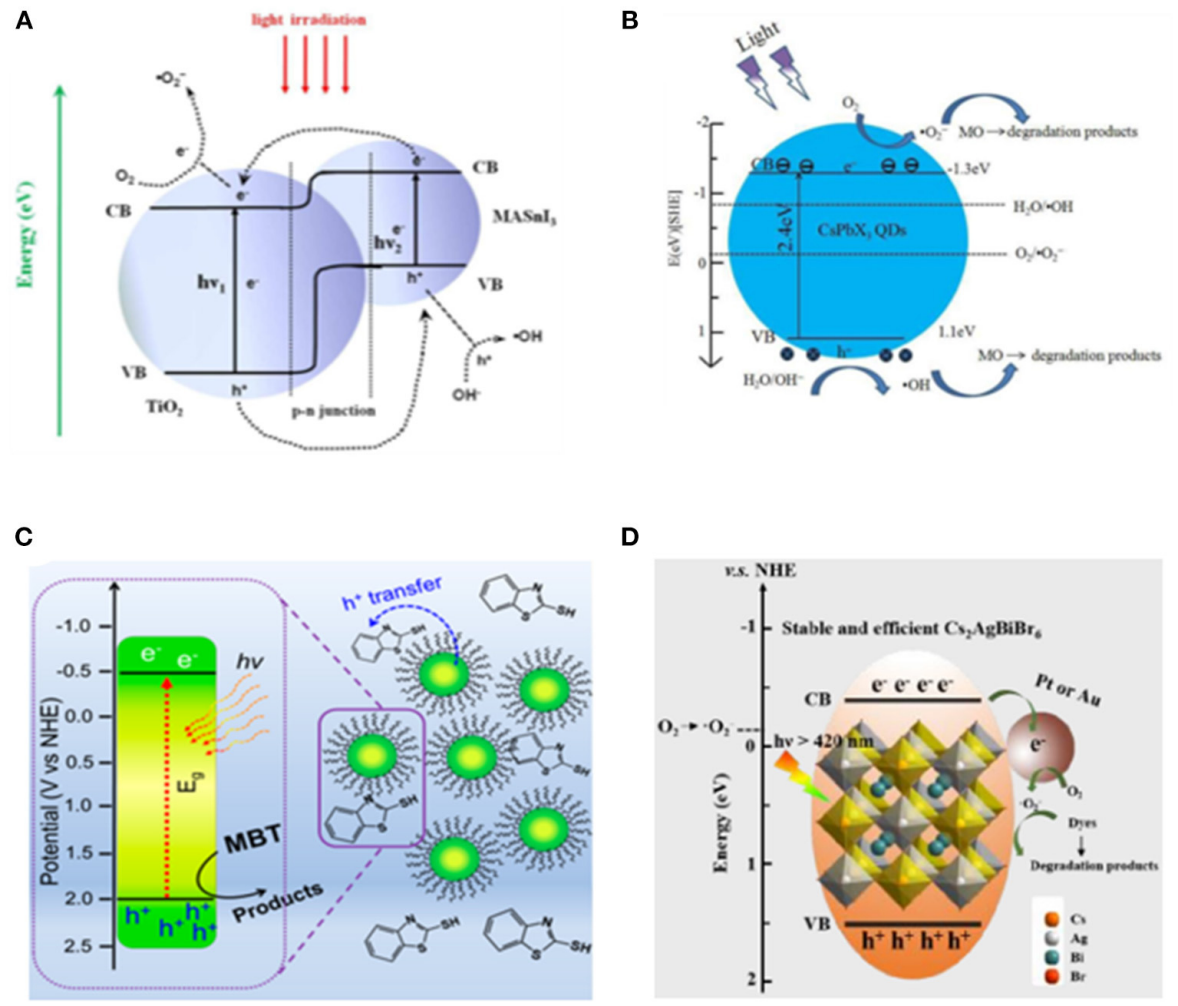
**(A)** Scheme of photoexcitation of the MASnI_3_/TiO_2_ (1:9) heterojunction composite and the subsequent photogenerated carrier transfer process. Reproduced by permission of The Royal Society of Chemistry. **(B)** Proposed mechanisms for the photocatalytic reaction of MO using CsPbX_3_ QDs. Reproduced by permission of The Royal Society of Chemistry. **(C)** Schematic diagram of dye degradation photocatalysis. Copyright © © 2019 American Chemical Society. **(D)** Schematic diagram of dye degradation with Cs_2_AgBiBr_6_ loaded with Pt or Au. © © 2019 Wiley-VCH Verlag GmbH & Co. KGaA, Weinheim.

Zhang et al. made the first trial using lead-free MASnI_3_-based heterojunction-type catalyst with TiO_2_ prepared via wet-chemical method for dye degradation (Zhang et al., 2017). By a rational design in band structure alignment, 97% of rhodamine B can be degraded within 40 min by the optimized MASnI_3_/TiO_2_ photocatalyst. The radical trapping experiment verifies the ${\text{O}}_{2}^{-}$ as the main active oxidizing species (Figure 16A).

Considering that the cations in organic–inorganic hybrid perovskite are likely to be simultaneously oxidized along with the target substrate, the all-inorganic halide perovskite is more favorable for this purpose. The CsPbX_3_ (X = Cl, Br, I) QDs have been utilized for photocatalytic degradation of methyl orange (MO) (Gao et al., 2017). They discovered that 90% of MO can be oxidized within 100 min when using CsPbCl_3_, compared with the 17% degradation yield in 80 min as a blank control with the same light source (Figure 16B).

A variety of chemicals have been investigated for degradation reaction. The 2-mercaptobenzothiazole (MBT) is a widely used intermediate product in industrial production with aquatic toxicity and poor biodegradability. CsPbBr_3_ QDs is employed to successfully degrade the MBT through photocatalytic and photoelectrocatalytic processes (Figure 16C). The degradation rate with perovskite QDs boosts by six times under visible light and almost doubles under UV-Vis light as well (Cardenas-Morcoso et al., 2019). Feng et al. prepared a CsPb(Br_1−x_Cl_x_)_3_-Au nano-heterostructure by reducing the Au on the CsPbBr_3_ surface using oleylamine (Feng et al., 2019). With this heterostructure as photocatalyst, a 71% of Sudan Red III can be degraded within 6 h compared with the 20% degradation yield obtained with pure CsPb(Br_1−x_Cl_x_)_3_ NCs. The double perovskite Cs_2_AgBiBr_6_ is also employed for dye degradation in the alcohol-based photocatalytic system (Zhang et al., 2019). A roughly 98% Rhodamine B can be degraded by Cs_2_AgBiBr_6_ within 120 min, whereas the Rhodamine 110, Methyl red, and Methyl orange show lower degradation rates (Figure 16D).

As previously mentioned, the design of a heterojunction structure turns out to be an effective strategy for efficient charge transport and highly catalytic surface. Zhao et al. built a Ag-CsPbBr_3_/CN photocatalyst by assembling nano-Ag, CsPbBr_3_ QDs, and bulk g-C_3_N_4_ (CN) (Zhao et al., 2019b). The 7%-Ag-CsPbBr_3_/CN composite displayed approximately 92.79% degradation rate of 7-aminocephalosporanic acid (7-ACA) within 140 min. Zhao et al. designed two types of heterojunction structures, CsPbBr_3_/CN (type I) and CsPbCl_3_/CN (type II), by *in situ* growth method. The degradation rate of penicillin 6-aminopenicillanic acid (6-APA) under visible light can reach 78.11 and 83.31% in 120 min, respectively (Zhao et al., 2019a).

The investigation on the perovskite-based photo-degradation AOP is still in infancy. Later studies should place more efforts on understanding the oxidation mechanism differing from the traditional titania-based photo-degradation. Overall, despite drawbacks of perovskite-based photocatalysis, the degradation of various organic contaminants has been studied in different mediums.

## Photocatalytic Synthesis of Organic Molecules

Photo-induced redox processes enable a variety of catalytic transformations in organic synthesis under visible light triggered by the sensitizing dyes (Teplý, 2011). As a photosensitive semiconductor, perovskite can be a replacement for sensitizing dyes in photoredox catalysis. Therefore, the perovskite photocatalytic synthesis of organic molecules has also emerged as a promising tool for chemical biology and materials chemistry. A variety of strategies have been employed to design the perovskite photoredox catalyst in organic chemistry.

### Lead-Free Perovskite

**Figure 17:**
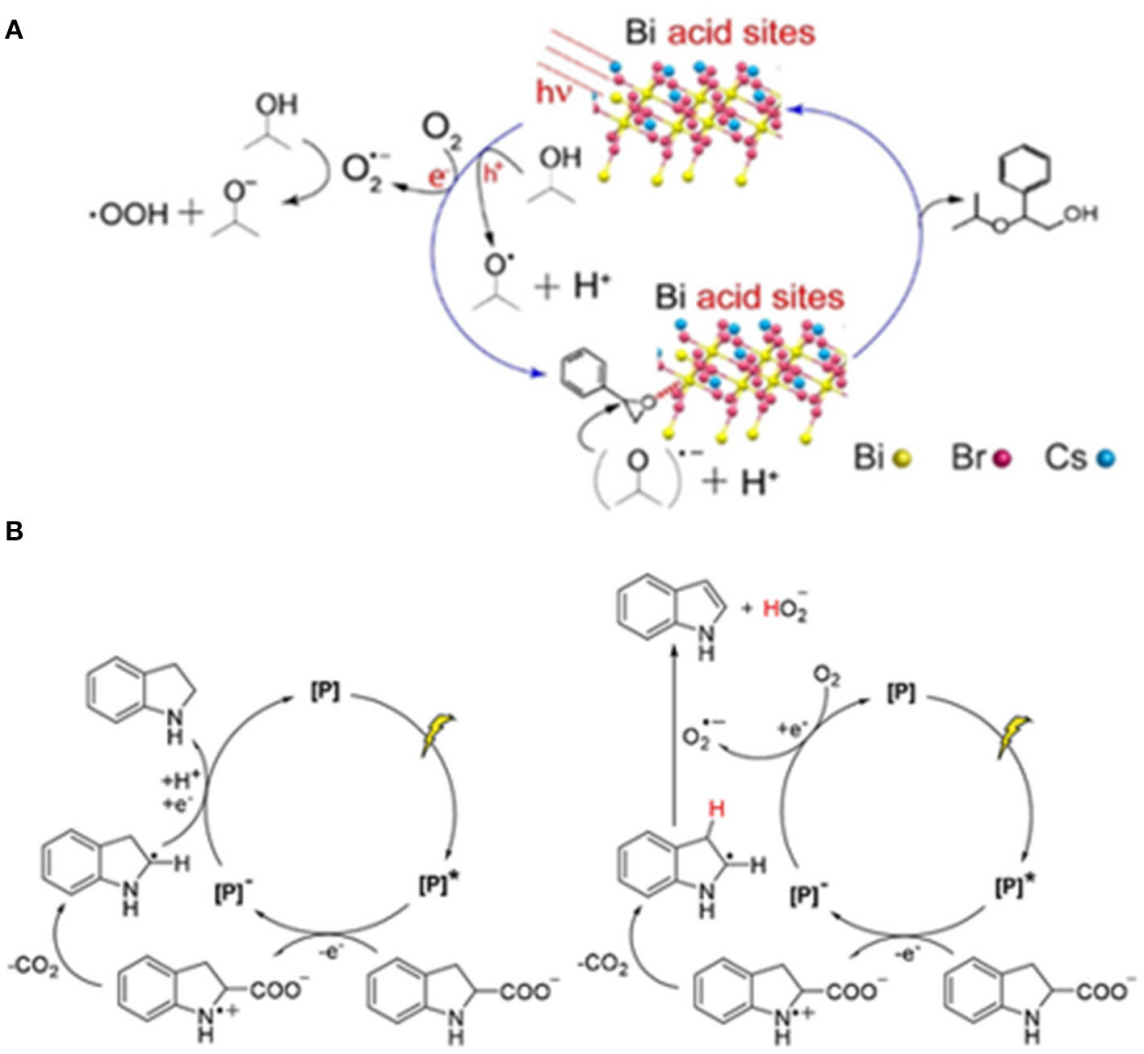
**(A)** Proposed photocatalytic cycle for epoxide alcoholysis reaction over Cs_3_Bi_2_Br_9_ with proper Lewis acid sites on surface. © © 2019 Wiley-VCH Verlag GmbH & Co. KGaA, Weinheim. **(B)** Proposed mechanisms for the decarboxylation (left) and dehydrogenation reactions (right). © © 2019 Wiley-VCH Verlag GmbH & Co. KGaA, Weinheim.

Dai et al. investigated the photocatalytic ring-opening reaction of epoxides activated on lead-free perovskite Cs_3_Bi_2_Br_9_ and obtained the value-added β-alkoxy alcohols (Figure 17A) through a nucleophilic addition between epoxides and alcohols, wherein the isopropanol serves as both solvent and reactant. Cs_3_Bi_2_Br_9_ show a drastically improved performance (86% yield, 1,333 μmol h^−1^ ${\text{g}}_{\text{cat}}^{-1}$) than CsPbBr_3_ (<1% yield). The reaction can be adapted to numerous alcohols including methanol, ethanol, 1-butanol, 2-butanol, *tert*-butanol, and even thiols. The key to the reaction lies on the acid sites on the Cs_3_Bi_2_Br_9_ surface, enabled by the Bi cations. Although the CsPbBr_3_ has the merit of high light absorption efficiency owing to the smaller band gap, the weak Lewis-acid sites on Pb-based perovskite leads to poor activity in ring-opening reactions (Dai and Tüysüz, 2019). This is by far the only report of lead-free perovskite-based photocatalytic chemical synthesis.

### 2D Perovskite in Photocatalysis

Hong et al. introduce hydrolytically stable 2D perovskite containing hydrophobic hexadecylammonium (HDA) cations as the intercalating layers in photocatalysis in order to overcome the instability of 3D perovskite, especially in protic solvent. Two photocatalysts (HDA)_2_PbI_4_ and (HDA)_2_SnI_4_ are successfully synthesized confirmed by the single-crystal X-ray diffraction. The (HDA)_2_PbI_4_ can maintain the green light emission under UV when suspended in water. The catalysts are used for photo-redox decarboxylation and dehydrogenation reactions. The reaction runs in two directions (Figure 17B): (I) Indoline-2-carboxylic acid undergo decarboxylation to form indoline under N_2_. (II) Both decarboxylation and dehydrogenation to indole in the presence of O_2_ (Hong et al., 2019).

### Oxidation Reaction

The photocatalytic degradation of organics shares the similar mechanism with oxidative synthesis. However, this part will focus on the different methods to obtain a particular product or achieve a particular reaction. The PEDOT, which is a conducting polymer widely used in optoelectronic devices, is usually prepared by the oxidation of 3,4-ethylenedioxythiophene. Chen et al. demonstrates that the 2,2′,5′,2″-Ter-3,4-ethylenedioxythiophene (TerEDOT) can be photocatalytically oxidized by the CsPbI_3_ perovskite QDs along with 4-benzoquinone (Qu) or molecular oxygen as the electron acceptor in toluene under visible light. The QDs are encapsulated in PEDOT polymer, which serves as a protecting layer to preserve the QDs from degradation and maintains the cubic structure (Chen et al., 2017).

Later, Wong et al. discovered that CsPbBr_3_ NCs could be used as an *in situ* photo-initiator for the polystyrene polymerization from vinyl monomers. The as-obtained polystyrene possesses a molecular weight of ~200 kDa and a polydispersity index (PDI) of ~2. The CsPbBr_3_ NCs are dispersed into a styrene monomer solution at 0.2 wt% concentration, which is then drop-casted and left to stand for 30 min under ambient conditions to dry off, forming a perovskite–polystyrene nanocomposite thin film. They postulate that the adsorption and coordination of the olefin to the perovskite surface could occur through the interaction between the filled π orbitals of olefin and the electron-deficient Pb^2+^, followed by an active radical mechanism to initiate the polymerization. The methodology can be further extended to the polymerization of poly(methyl methacrylate) (PMMA), with a conversion yield of 25% in 12 h (Wong et al., 2018).

The oxidation reaction of styrene is achieved by Qiu et al. This reaction is catalyzed by Zr species medicated and Cl-doped CsPbBr_3_/Cs_4_PbBr_6_ nanosheets. The surface modification with Zr species and Cl-doping are achieved simultaneously by using ZrCl_4_. A photocatalytic oxidation rate of styrene to benzaldehyde can be achieved at 1,098 μmol g^−1^ h^−1^, which is 2.9 times higher than that of pristine CsPbBr_3_/Cs_4_PbBr_6_ nanosheets (372 μmol g^−1^h^−1^) (Qiu et al., 2020).

Huang et al. synthesized perovskite/TiO_2_ heterostructure to achieve selective photocatalytic oxidation of benzylic alcohol to aldehyde. The optimized performance is achieved from the FAPbBr_3_/TiO_2_ with 96% selectivity to aldehyde. To prevent moisture damage to perovskite, the reaction is carried out in toluene by using molecular oxygen as electron acceptor (Huang et al., 2018a). Inspired by this heterojunction design, a series of CsPbBr_3_/TiO_2_ photocatalysts with different perovskite/TiO_2_ ratios are fabricated for photocatalytic oxidation reaction of toluene (Zhu et al., 2020). Toluene can be oxidized into benzyl alcohol and benzaldehyde by the catalysts. The optimal 6% perovskite/TiO_2_ sample shows the champion benzaldehyde production rate of 2,356 μmol g^−1^h^−1^.

Superfluous oxygen could damage the organic cation in perovskite (Aristidou et al., 2015). Schünemann et al. used all-inorganic CsPbBr_3_ instead of FAPbBr_3_ to fabricate a CsPbBr_3_/TiO_2_ nano-composite. This photocatalyst is also employed to the selective oxidation of benzyl alcohol to benzaldehyde under visible light (λ >420 nm). This reaction is carried out in a stainless-steel autoclave equipped with a borosilicate window with 5 ml of 0.1 M benzyl alcohol solution in toluene at 80°. The photocatalytic tests are performed in a range of solvents with various polarities, including cyclohexane, *n*-hexane, and α,α,α-trifluorotoluene, to investigate how solvent affects the activity and selectivity. The selectivity >99% can be achieved toward benzaldehyde at a conversion of 50% in cyclohexane. The conversion with plain P25 is almost independent of the solvent, whereas the conversion over CsPbBr_3_/P25 composite surprisingly increases as the solvent polarity decreases (Schünemann et al., 2018).

The NiO_x_ and related complexes have been widely studied in organic synthesis like aerobic epoxidation of alkenes, oxidative dehydrogenation of alkanes to alkenes, as well as photocatalytic organic transformation (Punniyamurthy et al., 2005). Inspired by the rational design in perovskite-based photovoltaic device, Huang et al. designed a ternary NiO_*x*_/FAPbBr_3_/TiO_2_ nanocomposite heterojunction for efficient activation of C(sp^3^)–H bonds in alkanes using molecular oxygen. For this hybrid structure, the TiO_2_ and NiO_x_ offer electron and hole extraction/transport, respectively, enabling an efficient carrier separation. This work corroborates the capability to oxidize the C(sp^3^)–H bond in alkane including toluene, cycloalkanes, and cyclooctane with over 99% selectivity (Huang et al., 2018b).

However, in oxidation reaction, the by-product, H_2_O, would induce damages to the perovskite lattice and limit the long-term photocatalysis of the reaction. Efficient removal of the water by-product via special reaction design could be a possible strategy for further practical application.

### C–C, C–O, and C–N Bond Formations

**Figure 18:**
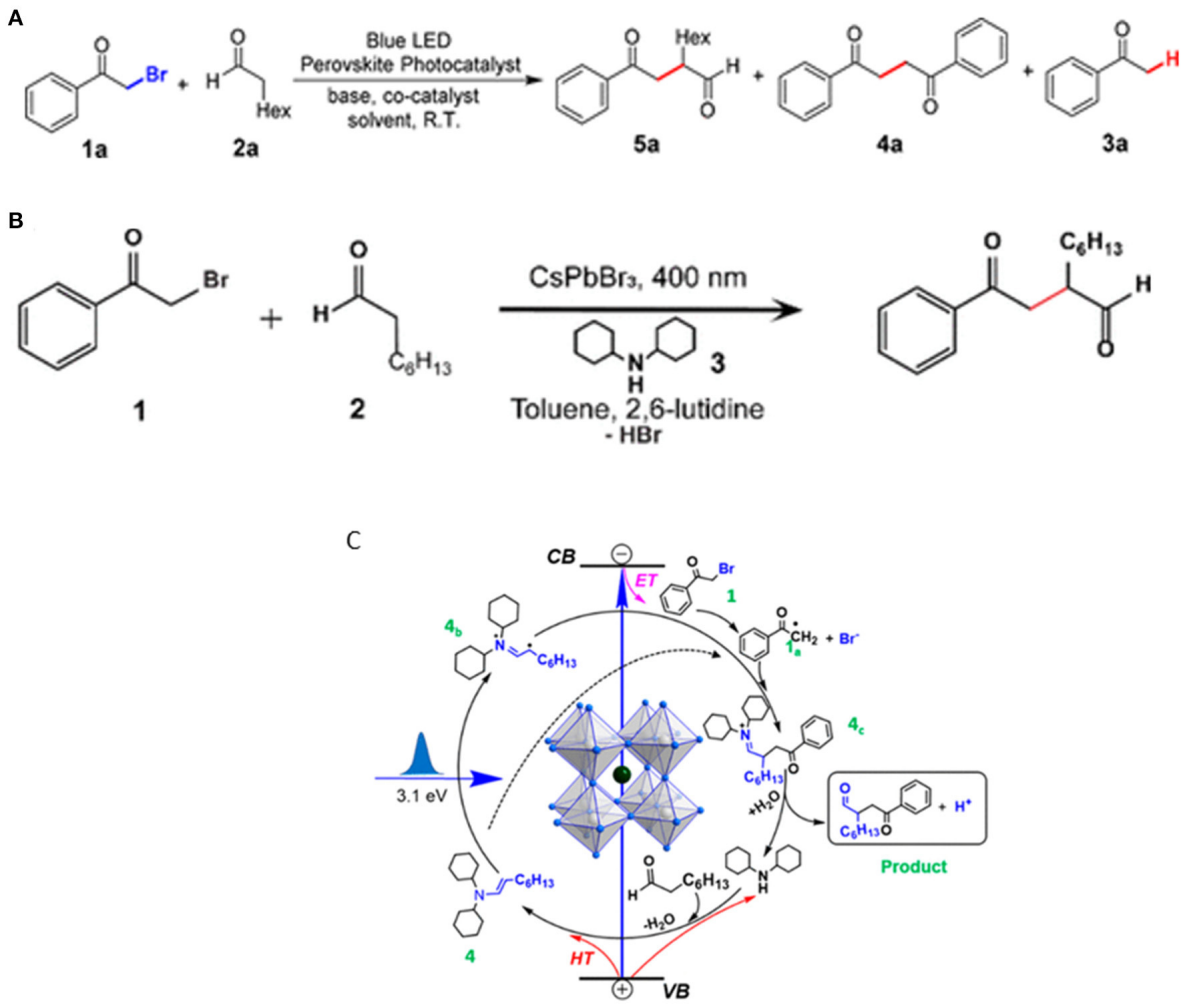
**(A,B)** α-Alkylation of aldehyde reaction. Copyright © © 2019, American Chemical Society. **(C)** Proposed reaction mechanism for α-alkylation catalyzed by CsPbBr_3_ NCs. Copyright © © 2020, American Chemical Society.

Nature is capable of transforming solar energy into chemical energy via the photo-induced formation of C–C, C–O, and C–N bonds starting from CO_2_ and light (Zhu et al., 2019a). Zhu et al. demonstrated that APbBr_3_ (A = Cs or MA)-type perovskite colloids can selectively photocatalyze the α-alkylation, and hence the carbon–carbon bond formation (Zhu et al., 2019b). The champion yield can reach up to 96% with optimized substrate ratio with the assistance of an amine cocatalyst (Figure 18A). This reaction is an elaborately selected photocatalysis system with the merits including cost-effectiveness, operational convenience, and practical scale-up adaptability, etc.

Wang et al. followed this work by studying the detailed mechanism of this reaction with ultrafast transient absorption spectroscopy (Wang K. et al., 2020). The model reaction is illustrated in Figure 18B between 2-bromoacetophenone and octanal by using dicyclohexylamine as a cocatalyst and 2,6-lutidine as a base. Combining the transient absorption spectroscopy result and energy band structure, it shows that the *in situ* formed enamine between the cocatalyst and 2-bromoacetophenone can accept the holes from the perovskite, and thus expedite the rate-determining step in this reaction. The charge separation state close to microsecond scale (~0.8 μs) allows the photo-generated charged radical intermediates to form a C–C bond through a bi-radical pathway Figure 18C.

**Figure 19:**
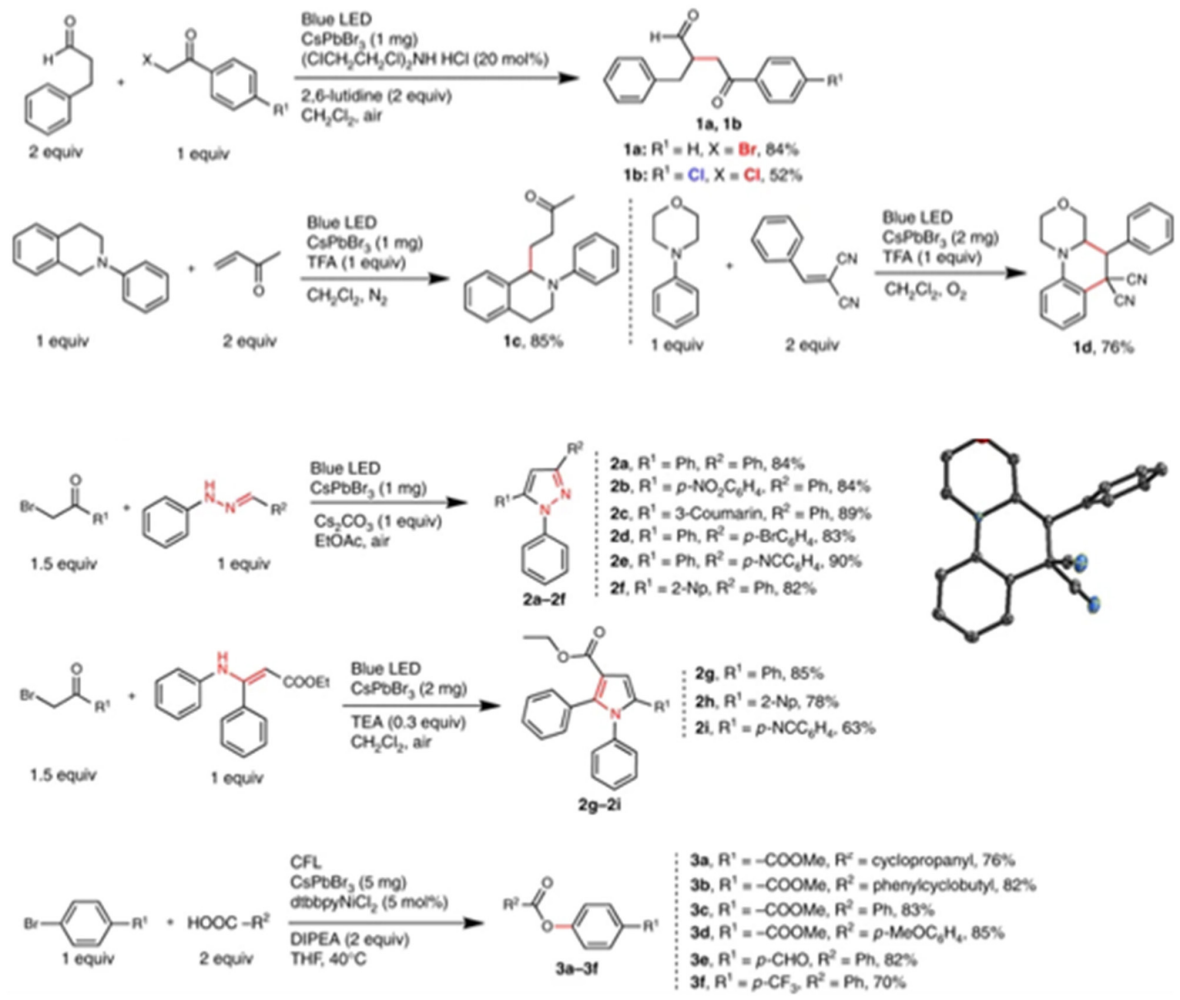
The library of C–C, C–N, and C–O bond formation reactions and respective yield.

Zhu et al. discussed some key concerns on perovskite as a photocatalyst in size, stability, reaction condition tolerance, and key catalytic metrics on the organic synthesis for successful C–C, C–O, and C–N bond formations. They discovered that there is a size effect on the reaction rate, wherein the smaller size distribution leads to a higher activity. The essential role of protons in these reactions is still unclear, but they may play a crucial role in organic synthesis. The perovskite NCs can be stabilized in non-halogen acid, but the appropriate reactions are only limited in a perovskite-friendly solvent environment (Figure 19; Zhu et al., 2019a).

**Figure 20:**
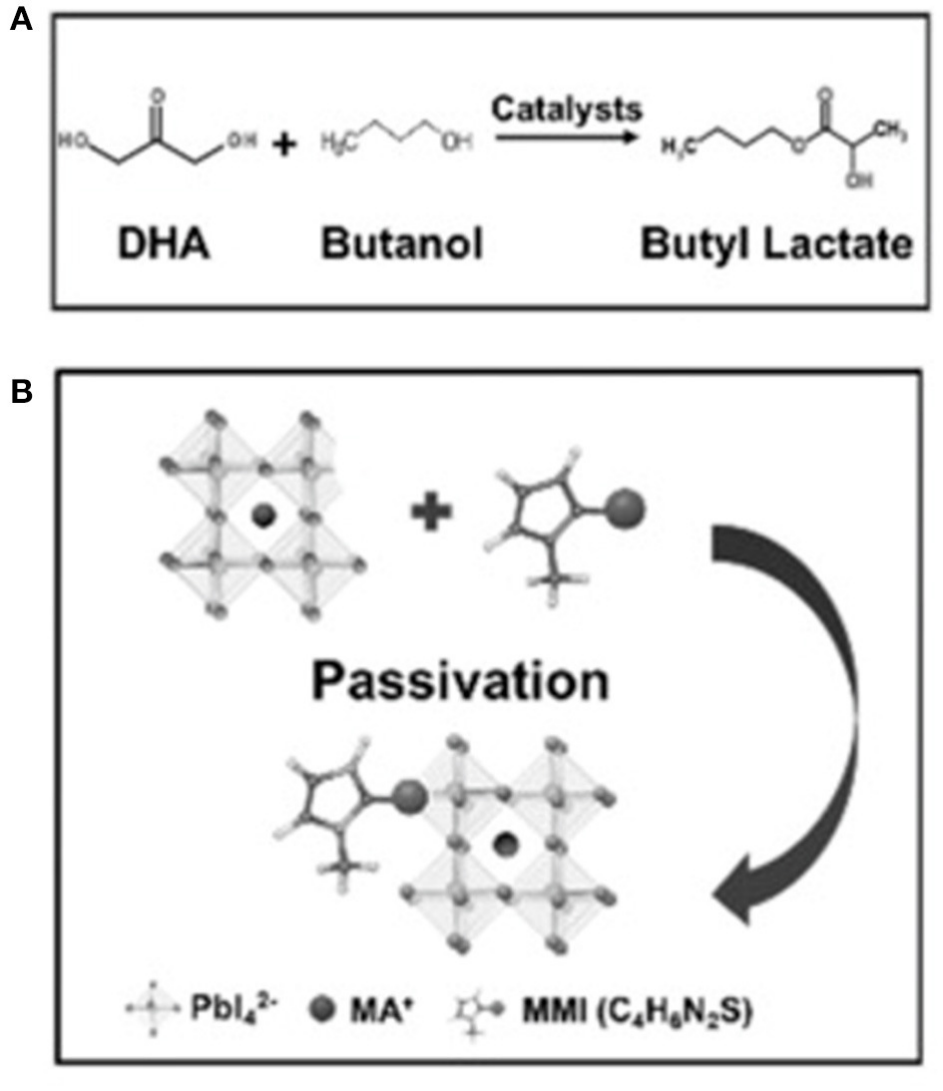
**(A)** The conversion of DHA and butanol into butyl lactate over the catalyst. **(B)** The passivation of MAPbI_3_ (Dong et al., 2020b) © 2020 Wiley-VCH Verlag GmbH & Co. KGaA, Weinheim .

Despite the above enumeration, Li et al. have reported a 1,3-dihydroxyacetone (DHA) conversion reaction catalyzed by MAPbI_3_. Figure 20A shows the photocatalytic synthesis of butyl lactate from DHA and butanol. The MAPbI_3_ photocatalyst exhibits a butyl lactate yield at 77 mg L^−1^. The desirable perovskite surface structure can be adjusted by exposing different terminating ions, i.e., MAI^−^ or (PbI_6)_ octahedron. By stripping off the I^−^ ions to form I vacancy, the Pb^II^ sites are verified as the effective binding site for this reaction, Therefore, the MAI-terminated surface with I^−^ vacancy shows better activity (Figure 20B). The MA^+^ cations and I^−^ in (PbI_6)_ octahedron are both proven to be catalytically inert (Dong et al., 2020b).

### Other Reactions

Nitrogen oxides (NO_x_) are one of the most dangerous air pollutants, which may cause acid rain. FAPbBr_3_ QDs is the first perovskite-based photocatalyst used for NO removal (Huo et al., 2020). The FAPbBr_3_ is considered as a potential candidate for NO removal. The silica sphere is employed to enhance their stability during the reaction. However, the NO degradation ratio gradually decreases in three cycles (70, 60, and 57% consecutively). During the first 30 min, the catalyst could still achieve 70% removal of NO under visible light at room temperature. The decrease in degradation rate is triggered by the competition between product NO_2_ and NO.

Wu et al. discovered that the thiophenol can be successfully coupled to form a disulfide in CH_2_Cl_2_ by using CsPbX_3_ (X = Cl, Br, I) QDs under white light. The best performance is accomplished with 1 mol% CsPbBr_3_ in a non-polar solvent system, CH_2_Cl_2_ or CH_2_Cl_2_/cyclohexane (1:1). The reaction could also be catalyzed by CsPbI_3_ or CsPbCl_3_ with lower activities. The mechanism involves the binding of thiols to the Pb(II) ions on the surface. First, the thiol group and the proton coordinate to the Pb(II) and halide ion, respectively. Upon light radiation, the electron generated in the perovskite transfers to the proton, while the mercaptan group absorbs the hole, creating a hydrogen radical, and a thiol radical. The presence of O_2_ may promote the thiol coupling (Wu et al., 2018a). They also discovered that the C–H activation in this reaction is a key step, which can be effectively triggered by CsPbX_3_ (X = Br, Cl, I) NCs. The yield rises from 50% up to 96%.

DuBose et al. proposed that the ferrocene redox couple can be used as redox probes to study the photocatalysis of CsPbBr_3_ NCs. They discovered that with the addition of high-concentration ferrocenium (Fc^+^) in the system, the emission of CsPbBr_3_ NCs would be quenched due to the reduction of Fc^+^ to ferrocene (Fc^0^), whereas a subsequent emission recovery is discovered at low Fc^+^ concentrations. The rate constant of the photo-induced interfacial electron transfer between CsPbBr_3_ and ferrocenium (Fc^+^) is determined to be 1.64 × 10^10^ s^−1^ using TA (DuBose and Kamat, 2019).

## Conclusion and Prospects

In this review, we have discussed the recent studies on photocatalytic applications of the halide perovskite-based materials from the following perspectives: (I) Design of photoelectrocatalytic device structures including the n-i-p/p-i-n structure, photoelectrode device encapsulation, and electrolyte engineering; (II) The design of heterojunction-type photocatalytic systems toward the hydrogen evolution reaction (HER) and CO_2_ reduction reaction, including light management, surface/interface engineering, stability improvement, product selectivity engineering, and reaction system engineering; and (III) The photocatalysts for the environmental application and organic synthesis. The unique properties of perovskite lead to higher requirements for reaction selection, reaction system design, and catalyst nano-structure design. The applications are focused on some particular reactions or molecular synthesis. Multiple strategies have been developed to improve the photocatalytic performance. A smart design may significantly improve carrier separation efficiency and stability at the same time.

The challenges in perovskite-based photoelectrocatalysis lie on the combination of active electrode materials and appropriate encapsulation. It has been proven that the perovskite surface is only catalytically active in some specific reactions (Zhu et al., 2019a). Meanwhile, the catalytic active site can also be relocated to another material surface through the heterojunction nano-structure design, which not only promotes the carrier separation but also may improve the stability and hence extends the application of the perovskite toward diversified catalytic processes.

We would expect some breakthrough in the following aspects in the future: stability improvement, insightful mechanistic understanding of connection and carrier's transport at the heterojunction interface, in-depth study on the role of the surface ligands, and construction of catalytically active site. Last but not the least, all the above aspects must be investigated and addressed in the context of reaction system design. We believe that halide perovskite-based photocatalytic system could potentially replace the traditional photocatalysts for some particular applications.

## Author Contributions

This manuscript was written through contributions of all authors. YZ, LW, and TS carried out most of the literature research, summary, and most of the writing. YL, AM, and QC designed the article structure and finalized the manuscript. All authors have given approval to the final version of the manuscript.

## Conflict of Interest

The authors declare that the research was conducted in the absence of any commercial or financial relationships that could be construed as a potential conflict of interest.
